# Quantitative structure–property relationship modeling and ranking of necrotizing fasciitis drugs via degree-based topological indices

**DOI:** 10.3389/fchem.2025.1668093

**Published:** 2025-10-28

**Authors:** A. Joy Prisca, B. Jaganathan

**Affiliations:** Department of Mathematics, School of Advanced Sciences, Vellore Institute of Technology, Chennai, India

**Keywords:** degree-based indices, necrotizing fasciitis, regression analysis, quantitative structure–property relationship, multi-criteria decision-making, technique for order of preference by similarity to ideal solution, multi-objective optimization on the basis of ratio analysis

## Abstract

**Introduction:**

The increasing incidence and high mortality rate of necrotizing fasciitis (NF), a rapidly progressing infection of the fascia and subcutaneous tissue, highlights the urgent need for effective drug evaluation strategies. Traditional clinical trials for NF antibiotics are costly and time-consuming, necessitating the development of computational approaches that can reliably capture drug behavior.

**Methods:**

The study employs degree-based topological indices to represent molecular structures of NF antibiotics and develops QSPR models to predict their physicochemical properties. Calculating topological indices, performing regression analyses to identify significant indices, and using these indices in multi-criteria decision-making techniques to rank the antibiotics.

**Results:**

This study demonstrates the potential of degree-based TIs combined with regression and multi-criteria decision-making techniques to predict and rank the physicochemical properties of antibiotics used to treat necrotizing fasciitis (NF).

**Discussion:**

This integrated approach demonstrates the utility of topological indices in predicting drug properties, prioritizing candidates, and supporting the rational design and repurposing of NF therapeutics.

## 1 Introduction

Necrotizing fasciitis (NF) is an uncommon bacterial infection of the skin and underlying tissues that has a high risk of death and disability (Tantirat et al., 2020). The disease was first discovered in 1783 in France and occurred sporadically throughout the 19th and 20th centuries (Misiakos et al., 2014). In the period following COVID, there has been a notable increase in the fatality rate of streptococcal necrotizing fasciitis, with an increase in the mortality rate of approximately 13%–18% (da Costa Senior et al., 2024). The literature shows that the average mortality rate varies between 25% and 73%. Additionally, the prevalence of invasive NF has increased in Europe, Japan (Choudhary et al., 2024), and Thailand. The incidence of NF is approximately fifteen cases per hundred thousand individuals worldwide. Furthermore, NF can severely impair the quality of life of patients, especially those with limb loss. Thus, research is of paramount importance because of the high death rate, rising number of cases, high expense of treating NF, and the noticeable drop in the quality of life after treatment (R Vignesh et al., 2024).

Infections acquired through cuts, scrapes, burns, insect bites, piercing bruises, intravenous medication use, surgical cuts, sores that come into contact with fresh or salt water, and consumption of contaminated oysters can potentially develop into NF (Yu et al., 2022). There are case studies in which other infections, such as chicken pox, monkey pox, and viral infections, complicated NF (Lovis et al., 2023; Moshfegh et al., 2025; Zhang et al., 2023). Even people in good health can develop type 1 NF, which is brought on by a polymicrobial infection. Furthermore, obligatory airborne organisms and fungi were identified as the causative factors of NF. This provides more rapid answers to the overburdened healthcare situation and medicinal requirements, thus significantly reducing the time required (Kulkarni et al., 2023).

NF remains a severe clinical burden because of its rapid progression, high case fatality rate, and the frequent involvement of multidrug-resistant pathogens. Although broad-spectrum agents such as piperacillin, vancomycin, and imipenem are the mainstay of therapy, their clinical efficacy is often hampered by diagnostic delays, inadequate penetration of drugs into necrotic tissue, and resistance mechanisms in organisms such as *Staphylococcus aureus* and *Pseudomonas aeruginosa*. Traditional laboratory-based evaluations of antibiotic effectiveness are costly, labor-intensive, and unsuitable for urgent therapeutic decision-making, highlighting the importance of computational methods. Among these, quantitative structure–property relationship (QSPR) modeling is widely recognized as a powerful approach in drug research. By linking molecular descriptors to physicochemical and pharmacokinetic attributes, QSPR facilitates the prediction of drug performance, reduces the reliance on experimental screening, accelerates discovery pipelines, and deepens the understanding of structure–property relationships that are essential for rational drug design and repurposing. Topological indices (TIs) are essential in QSPR investigations. TIs help anticipate a compound’s potential for therapeutic use by using mathematical correlations to associate its chemical composition with its biological activity. TIs are mathematical illustrations that reflect the geometric and topological properties of molecular structures and provide vital information regarding pharmacological interactions (Anuradha and Jaganatha, 2024; Pattabiraman and Cancan, 2023; Anuradha et al., 2024; Harshavardhan et al., 2025). TIs provide crucial information about the stereochemistry of substances by focusing on their spatial structure, symmetry, and molecular connectivity (Suresh et al., 2024). Recently, TIs have been used in cancer medicine repurposing strategies (Yousaf and Shahzadi, 2024).

**FIGURE 1:**
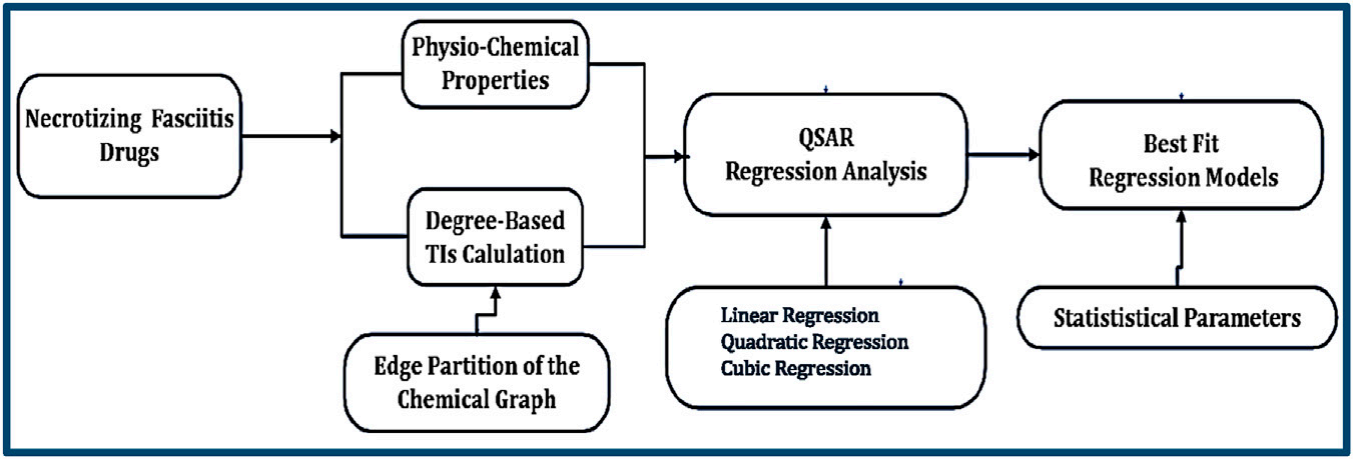
Process of identifying the best-fit regression model.

In this study, various valency-based TIs were calculated, and a large dataset was built and validated to create reliable QSPR models. The workflow of the process is shown in Figure 1.

By analyzing a large dataset that links TIs to the physicochemical characteristics of NF medicines, reliable QSPR models were created in this study. Several medications with possible antibiotic qualities were highlighted by the strong prediction ability of the processes. Linear, quadratic, and cubic regression models were used to assist research workers in understanding the structural design of antibiotic NF drugs and support the ability to work with molecular structures.

Drug discovery requires balancing multiple molecular features, namely, solubility, lipophilicity (logP), molecular weight, topological polar surface area (TPSA), bioavailability, and toxicity, which often conflict because high binding affinity may coincide with poor pharmacokinetics. Multi-criteria decision-making (MCDM) methods, including the technique for order of preference by similarity to ideal solution (TOPSIS) and multi-objective optimization on the basis of ratio analysis (MOORA), resolve this complexity by normalizing diverse descriptors, applying criterion-specific weights, and producing composite rankings. When integrated with regression-based QSPR modeling, these approaches enable systematic prioritization of neem phytochemicals, improving efficiency in lead identification and guiding rational drug optimization (Anuradha and Jaganathan, 2025; Kanwal et al., 2023).

## 2 Materials and methods

### 2.1 Antibiotics for necrotizing fasciitis

Necrotizing fasciitis, sometimes referred to as flesh-eating sickness, is caused by a bacterial infection that damages the fascia, the tissue beneath the skin. Comprehensive debridement (surgical removal of necrotizing tissue) and empiric antibiotics are essential treatments that may prevent aggressive progression without causing death. Antibiotics with a wide spectrum should be prescribed to patients as soon as NF is diagnosed. Antibiotics are empirically used depending on the microbial categorization of the NF. Soft tissue reconstruction is a feasible choice after all the afflicted tissues have been debrided. Comprehensive treatment of NF involves careful administration of antibiotics.

Antibiotics such as piperacillin, vancomycin, imipenem, and daptomycin remain central to NF therapy because of their broad-spectrum activity against Gram-positive and Gram-negative bacteria, toxin suppression, and efficacy against resistant strains. Clindamycin is often used as an adjunct to inhibit toxin production, whereas ertapenem and gentamicin serve as alternatives for penicillin-allergic patients or for those with Gram-negative infections. The early use of these drugs reduces mortality, tissue necrosis, and the need for surgical interventions, especially in vulnerable populations. Given NF’s rapid progression, high fatality, and multidrug resistance, degree-based topological indices (TIs) with QSPR regression provide a cost-effective strategy to predict drug properties. Coupled with MCDM, this framework supports rational antibiotic prioritization and optimizes therapeutic decisions (Bisgaard and Bulger, 2024; Jain et al., 2023).

**FIGURE 2.**
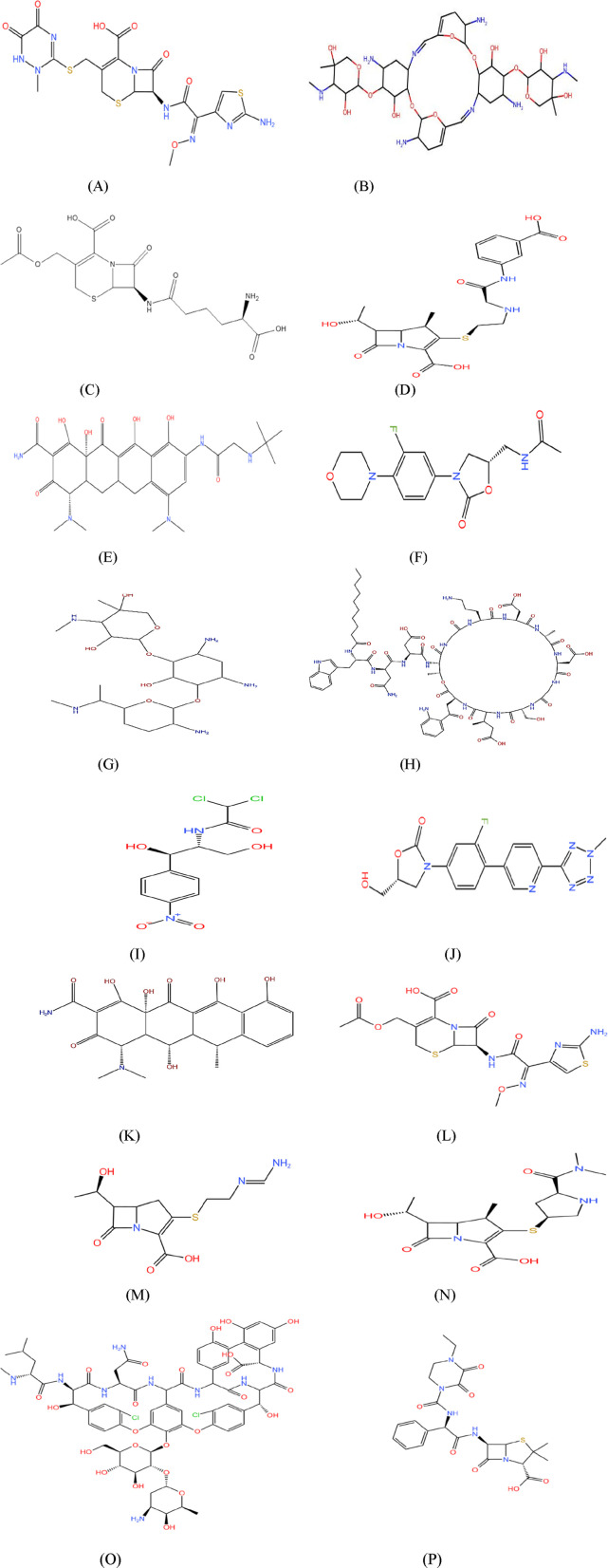
Molecular structure of NF drugs: **(A)** ceftriaxone, **(B)** aminoglycoside 66-40C, **(C)** cephalosporin, **(D)** ertapenem, **(E)** tigecycline, **(F)** linezolid, **(G)** gentamicin, **(H)** daptomycin, **(I)** chloramphenicol, **(J)** tedizolid, **(K)** doxycycline, **(L)** cefotaxime, **(M)** imipenem, **(N)** meropenem, **(O)** vancomycin, and **(P)** piperacillin.

In this study, several valency-based TIs were used to investigate the antibiotics used for treating NF. The spatial configurations of the atoms within the molecules were studied using topological analysis and stereochemistry. The chemical structures were drawn using KingDraw with molecular data obtained from PubChem and ChemSpider (Figure 2).

### 2.2 Topological indices (degree-based)

A molecular descriptor derived from the topology of a chemical compound is called a TI (Anuradha and Jaganathan, 2025). The relationship among a molecule’s structure, chemical reactivity, and physical properties can be calculated using the logical and mathematical techniques of graph theory (Anuradha and Jaganathan, 2023; Joy Prisca and Jaganathan, 2025). Degree-based topological indices (TIs), such as Randić, Zagreb, ABC, and discrete Adriatic indices, were selected for their established utility in QSPR studies. The Randić index captures molecular branching, the Zagreb indices characterize stability and connectivity, and the ABC index effectively models thermodynamic and physicochemical properties. Discrete Adriatic indices provide additional sensitivity to the structural complexity. Recent studies have confirmed their predictive reliability for modeling alkaloids and quinolone antibiotics (Kirana et al., 2024; Pulakos et al., 2000). Together, these indices form a computationally efficient and theoretically robust framework for investigating the physicochemical properties of drugs used to treat NF. Degree-based TIs have certain benefits over other indices, such as those based on distance, entropy, or temperature. These advantages include ease of calculation, direct connection to the local connectivity of atoms, and strong associations with chemical and physical properties (Mahboob et al., 2024). These descriptors provide the relationship between the natural physical characteristics of atoms and their positions in a chemical graph structure as a polynomial or number (Baby et al., 2023; Zhao et al., 2021). Every atom and bond between them are conceptualized as the vertices and edges of the mathematical graph. The degree of any vertex is the quantity of (edges) bonds that are incident on it (atoms). This degree represents the valence of any atom. Many chemical structures have been analyzed using degree-based indices (Murugan et al., 2023; Tharmalingam et al., 2023; Yang et al., 2023).

Throughout this article, o represents any chemical graph, $V\left(o\right)\text{ is}$ the set of all vertices $\left\{v,v_{1},v_{2}\ldots \right\}$ , and $E\left(o\right)$ represents the collection of all edges of $\left\{S{,S}_{1},S_{2},S_{3}\ldots \right\}$ . Two vertices, v and $v_{i}$ , are said to be neighboring if there exists an edge to link them. Let $S=vv_{1}$ represent an edge in the graph $E\left(o\right)$ with ends at v and $v_{1}$ . The number of edges incident on any vertex v is the degree of the vertex and is denoted as $d_{v}$ . All the TIs considered for the study are degree-based indices that use the edge-partition technique. In the molecular structure of a chemical compound, overvaluation extends to all edge partitions.

Many physicochemical characteristics are needed for drug-likeness prediction, especially the ADMET (absorption, digestion, metabolism, excretion, and toxicity) properties governed by Lipinski’s rule of five (Abdullah et al., 2023). Molecular mass, octanol–water partition coefficient, molar refractivity, enthalpy of vaporization, and polarizability are a few examples of physicochemical properties in the list. Many of these properties can be predicted by TIs, and a few are narrated for better understanding. Degree-based topological indices were selected for studying antibiotics for NF because they directly quantify atomic connectivity and branching, which strongly influence the physicochemical descriptors summarized in Tables 1, 2. Properties such as molecular weight, TPSA, polar surface area, polarizability, molar refractivity, and complexity are inherently related to how atoms are arranged and bonded within the molecular graph (Shanmukha et al., 2020). These indices offer a computationally efficient way to capture these structural features, enabling reliable QSPR modeling and regression analysis to predict and rationalize the drug properties critical for NF therapy. For example, the first and second Zagreb indices are applied in the study of the complexity and molecular chirality of chemical compounds (Saidi et al., 2020). In a study of the heat of formation in octanes and heptanes, the augmented Zagreb index was a suitable predictive index. The heat of formation, stability, and strain energy of alkanes and cycloalkanes are all highly correlated with the ABC index. The Randić index and its derivative, the sum-connectivity index, correlate well with the π-electronic energies of benzenoid hydrocarbons. Hence, TIs are widely used to investigate the properties of drug molecular structures (Adnan Mr et al., 2022; Kirmani et al., 2021; Mondal et al., 2021; Pratheeba and Aarthi, 2025). Among the few degree-based indices chosen, which exhibit good correlation with experimental results, the prediction can be validated using correlation and regression models (Balasubramaniyan and Chidambaram, 2023; Huang et al., 2023; Kansal et al., 2023; Shi et al., 2025; Zaman et al., 2025). The TIs used in the study for the physicochemical prediction are listed along with their notation and formulation.

**TABLE 1:** Physiochemical properties of NF medications.

S. no	Drug compound	MW	Mass	TPSA	Complexity	BP	EV
1	Ceftriaxone	554.6	554.04	288	1,110	—	—
2	Aminoglycoside 66-40C	857	856.454	348	1,500	1,109.1	184.4
3	Cephalosporin C	415.4	415.1	202	737	814.7	128.7
4	Ertapenem	475.5	475.14	182	893	813.9	124
5	Tigecycline (4R)	585.6	585.28	206	1,240	890.9	135.7
6	Linezolid	337.35	337.143	71.1	472	585.5	87.5
7	Gentamicin	477.6	477.31	200	636	669.4	112.6
8	Daptomycin	1,620.7	1,619.71	702	3,480	2,078.2	366.9
9	Chloramphenicol	323.13	322.01	115	342	644.9	100
10	Tedizolid	370.3	370.12	106	543	614.5	95.9
11	Doxycycline	444.4	444.15	182	956	762.6	116.5
12	Cefotaxime	455.5	455.05	227	833	—	—
13	Imipenem	299.35	299.09	142	491	530.2	92.7
14	Meropenem	383.5	383.15	136	679	627.4	106.4
15	Vancomycin	1,449.2	1,447.43	531	2,960	—	—
16	Piperacillin	517.6	517.16	182	982	—	—

**TABLE 2:** Physiochemical properties of NF medications.

S. no	Drug compound	FP	MR	PSA	Polarization	St	MV
1	Ceftriaxone	—	130	288	51.5	100.1	281.7
2	Aminoglycoside 66-40C	624.6	196.3	348	77.8	72.4	491.1
3	Cephalosporin C	446.5	96	202	38.1	82.2	266.6
4	Ertapenem	446	118.3	182	46.9	84.9	306.2
5	Tigecycline (4R)	492.6	151.3	206	60	80	402.5
6	Linezolid	307.9	83	71	32.9	47.7	259
7	Gentamicin	358.6	122.6	200	48.6	62.8	366.9
8	Daptomycin	1,210.7	399.3	702	158.3	85.3	1,110.8
9	Chloramphenicol	343.8	72.6	115	28.8	66.1	208.8
10	Tedizolid	325.4	93.6	106	37.1	62.3	235.6
11	Doxycycline	415	109	182	43.2	99.2	271.1
12	Cefotaxime	—	106	227	42	81.2	252.8
13	Imipenem	274.5	72.7	139	28.8	71	183.9
14	Meropenem	333.2	96.8	135	38.4	68.4	268.9
15	Vancomycin	—	350.8	530	139.1	105.1	874.7
16	Piperacillin	—	128.5	182	50.9	80.6	340.5

The sum connectivity index (SC) is formulated as follows:

$$\sum_{S\epsilon E\left(o\right)}\frac{1}{\sqrt{d_{v}+d_{v_{1}}}}.$$

The atom-bond connectivity (ABC) index is formulated based on the connectivity between the atoms proposed by Estrada et al., as follows:

$$\sum_{S\epsilon E\left(o\right)}\sqrt{\frac{d_{v}+d_{v_{1}}-2}{d_{v}d_{v_{1}}}}.$$

The first Zagreb index (M_1_) and the second Zagreb index (SZ) are among the elementary indices established by Trinajstic and Gutman, which is defined, respectively, as

$$\sum_{S\epsilon E\left(o\right)}d_{v}+d_{v_{1}}\text{ and }\sum_{S\epsilon E\left(o\right)}d_{v}\times d_{v_{1}}.$$

The second modified Zagreb index (M_2_) is defined as

$$\sum_{S\epsilon E\left(o\right)}\frac{1}{d_{v}\times d_{v_{1}}}.$$

The following is the formulation of the topological descriptors, SK and ${SK}_{1}$ index, for a molecular graph as defined by Shenghali and Kanabur, respectively:

$$\sum_{S\epsilon E\left(o\right)}\frac{d_{v}+d_{v_{1}}}{2}\text{ and }\sum_{S\epsilon E\left(o\right)}\frac{d_{v}\times d_{v_{1}}}{2}.$$

A topological index known as the geometric arithmetic index (GA) was developed with the following formulation:

$$\sum_{S\epsilon E\left(o\right)}\frac{2\;\sqrt{d_{v}\times d_{v_{1}}}}{d_{v}+d_{v_{1}}}.$$

The Randic index (Büyükköse and Cangul, 2022; Cruz et al., 2022) is formulated as

$$\sum_{S\epsilon E\left(o\right)}\frac{1}{\sqrt{d_{v}\times d_{v_{1}}}}.$$

The hyper Zagreb index (HZ) was defined for any simple connected graph, as follows:

$$\sum_{\left.\;S\epsilon E\left(o\right)\right)}{\left[d_{v}+d_{v_{1}}\right]}^{2}.$$

The Sombor index (SO) is a recently proposed molecular structure descriptor based on vertex degrees. It is defined as follows (Amin et al., 2021; Cruz et al., 2021; Gutman and Redžepović, 2022):

$$\sum_{S\epsilon E\left(o\right)}\sqrt{{d_{v}}^{2}+{d_{v_{1}}}^{2}}.$$

### 2.3 Bond additive topological indices

A degree-based (bond-additive) descriptor Des can be written as

$$Des\left(o\right)=\sum_{S\epsilon E\left(o\right)}F\left(d_{S\;}\left(v\right),d_{S\;}\left(v_{1}\right)\right).$$

Here, F is a mapping that gives a real estimate of the ordered pair, and $d_{v}\text{ and }{d_{v}}_{1}$ are the degrees of vertices v and $v_{1}$ , respectively, joining the edge S. In this study, a few Adriatic indices are used based on the characteristics they describe. The indices, along with their formulations and abbreviations, are provided in the following section (Vukicevic and Gasperov, 2010).

The inverse sum indeg index is defined by (exhibiting a good correlation with heat capacity)

$$IS\left(o\right)=\sum_{S\epsilon E\left(o\right)}\left(\frac{d_{v}d_{v_{1}}}{d_{v}{+d}_{v_{1}}}\right).$$

The misbalance indeg index is defined by (exhibiting a good correlation with enthalpy of vaporization)

$$MIR\left(o\right)=\sum_{S\epsilon E\left(o\right)}\left|\frac{1}{\sqrt{d_{v}}}-\frac{1}{\sqrt{d_{v_{1}}}}\right|.$$

The misbalance Rodeg index is defined by (exhibiting a good correlation with standard enthalpy of vaporization)

$$MR\left(o\right)=\sum_{S\epsilon E\left(o\right)}\left|\sqrt{d_{v}}-\sqrt{d_{v_{1}}}\right|.$$

The misbalance Deg index is defined by (exhibiting a good correlation with standard enthalpy of vaporization)

$${MD}^{2}\left(o\right)=\sum_{S\epsilon E\left(o\right)}\left|d_{v}-d_{v_{1}}\right|.$$

The misbalance Hadeg index is defined by (exhibiting a good correlation with standard enthalpy of vaporization)

$$MH\left(o\right)=\sum_{S\epsilon E\left(o\right)}\left|{\left(\frac{1}{2}\right)}^{d_{v}}-{\left(\frac{1}{2}\right)}^{d_{v_{1}}}\right|.$$

The min–max Rodeg index is defined by (exhibiting a good correlation with enthalpy)

$$M^{2}DR\left(o\right)=\sum_{S\epsilon E\left(o\right)}\sqrt{\frac{\min\left\{d_{v},d_{v_{1}}\right\}}{\max\left\{d_{v},d_{v_{1}}\right\}}}.$$

The max–min Rodeg index is defined by (exhibiting a good correlation with density)

$$M^{2}D^{2}R\left(o\right)=\sum_{S\epsilon E\left(o\right)}\sqrt{\frac{\max\left\{d_{v},d_{v_{1}}\right\}}{\min\left\{d_{v},d_{v_{1}}\right\}}}.$$

The max–min Deg index is defined by (exhibiting a good correlation with relative retention time)

$$M^{2}D^{2}\left(o\right)=\sum_{S\epsilon E\left(o\right)}\frac{\max\left\{d_{v},d_{v_{1}}\right\}}{\min\left\{d_{v},d_{v_{1}}\right\}}.$$

The max–min Sdeg index is defined by (exhibiting a good correlation with relative retention time)

$$M^{2}SD^{2}\left(o\right)=\sum_{\left.S\epsilon E\left(o\right)\right)}{\left(\frac{\max\left\{d_{v},d_{v_{1}}\right\}}{\min\left\{d_{v},d_{v_{1}}\right\}}\right)}^{2}.$$

The symmetric division Deg index is defined by (exhibiting a good correlation with TSA)

$$SD^{2}\left(o\right)=\sum_{S\epsilon E\left(o\right)}\left(\frac{\min\left\{d_{v},d_{v_{1}}\right\}}{\max\left\{d_{v},d_{v_{1}}\right\}}+\frac{\max\left\{d_{v},d_{v_{1}}\right\}}{\min\left\{d_{v},d_{v_{1}}\right\}}\right).$$

The physicochemical properties of the molecular structures considered in this study were obtained from ChemSpider and PubChem, as mentioned in Tables 1, 2.

The edge partition technique was used to calculate the aforementioned valency-based indices. The edge partitions of the NF medications mentioned in Figure 2 are presented in Table 3.

**TABLE 3:** Edge partition of the NF antibiotics.

S. no	Drug compound	$E\left(1,2\right)$	$E\left(1,3\right)$	$E\left(1,4\right)$	$E\left(2,2\right)$	$E\left(2,3\right)$	$E\left(2,4\right)$	$E\left(3,3\right)$	$E\left(3,4\right)$
1	Ceftriaxone	1	8	0	4	15	0	11	0
2	Aminoglycoside 66-40C	2	8	4	4	28	2	14	2
3	Cephalosporin C	0	9	0	4	8	0	8	0
4	Ertapenem	0	9	0	6	8	0	11	0
5	Tigecycline	0	12	4	1	7	1	17	3
6	Linezolid	2	4	0	6	12	0	4	0
7	Gentamicin	2	6	2	2	13	1	8	1
8	Daptomycin	3	29	0	16	51	0	17	0
9	Chloramphenicol	1	6	0	2	7	0	4	0
10	Tedizolid	1	3	0	4	17	0	5	0
11	Doxycycline	0	11	1	2	16	0	2	3
12	Cefotaxime	1	7	0	4	11	0	9	0
13	Imipenem	1	5	0	4	3	0	8	0
14	Meropenem	0	9	0	1	6	0	12	0
15	Vancomycin	2	23	2	3	51	1	27	1
16	Piperacillin	1	7	2	5	10	1	12	1

Tables 4–6 show the predictions made using the aforementioned edge partition and degree-based indices of the compounds used for NF treatment.

**TABLE 4:** Degree-based Adriatic TIs of NF medications.

S. no	Drug compound	IS	MIR	MR	MD^2^	MH	M^2^DR	M^2^D^2^R
1	Ceftriaxone	44.5	5.620	11.038	32	8.333	32.573	48.642
2	Aminoglycoside 66-40C	75.229	10.169	21.292	64	14.667	52.041	82.116
3	Cephalosporin C	32.35	4.842	9.131	26	7.333	37.386	37.386
4	Ertapenem	38.85	4.842	9.131	26	7.333	42.386	42.386
5	Tigecycline	53.576	8.419	16.399	48	12.667	60.236	60.236
6	Linezolid	29.4	3.248	6.742	20	4.667	35.625	35.625
7	Gentamicin	40.081	6.093	12.206	36	9	45.711	45.711
8	Daptomycin	126.45	19.753	38.682	112	29.333	149.934	149.934
9	Chloramphenicol	21.567	3.737	7.031	20	5.667	26.380	26.380
10	Tedizolid	34.817	3.767	8.014	24	5.333	36.431	36.431
11	Doxycycline	38.3929	7.457	14.942	44	11	48.113	48.113
12	Cefotaxime	36.617	4.679	9.035	26	7	40.011	40.011
13	Imipenem	24.017	2.795	5.028	14	4.333	11.241	25.749
14	Meropenem	32.95	4.582	8.495	24	7	35.937	35.937
15	Vancomycin	127.931	18.209	36.729	108	26.667	141.697	141.697
16	Piperacillin	45.564	5.833	11.571	34	8.667	49.355	49.355

**TABLE 5:** Degree-based TIs of NF medications.

S. no	Drug compound	M^2^D^2^	M^2^SD^2^	SD^2^	SC	ABC	M_1_	M_2_
1	Ceftriaxone	63.5	124.75	91.667	17.776	28.007	192	7.889
2	Aminoglycoside 66-40C	110.667	236.556	154.5	28.753	46.076	324	12.306
3	Cephalosporin C	51	111	71.333	13.344	21.167	140	6.222
4	Ertapenem	56	116	81.333	15.568	24.581	166	7.056
5	Tigecycline	86.5	215.083	116.917	19.902	32.896	236	8.681
6	Linezolid	44	77	67.333	13.633	21.327	148	5.722
7	Gentamicin	62.833	139.0278	86.25	15.915	25.338	174	7.264
8	Daptomycin	202.5	420.75	280.667	53.980	84.509	546	25.556
9	Chloramphenicol	36.5	79.75	49.6667	9.341	14.637	94	4.611
10	Tedizolid	45.5	78.25	67.333	13.721	21.339	146	5.889
11	Doxycycline	69	160.3333	89.833	16.053	25.845	170	7.556
12	Cefotaxime	52.5	104.75	75.667	14.671	23.029	156	6.667
13	Imipenem	33.5	67.75	49.667	9.685	15.073	102	4.556
14	Meropenem	49	107.5	69	12.582	20.298	142	5.583
15	Vancomycin	190.833	397.528	265.25	49.666	79.462	550	21.625
16	Piperacillin	66.333	144.278	94.583	17.629	28.114	196	7.792

**TABLE 6:** Degree-based TIs of NF medications.

S. no	Drug compound	SZ	SK	SK_1_	GA	Randic	HZ	SO
1	Ceftriaxone	231	96	115.5	37.568	17.116	972	139.6
2	Aminoglycoside 66-40C	394	162	197	61.313	27.415	1,684	236.873
3	Cephalosporin C	163	70	81.5	27.633	13.129	696	102.56
4	Ertapenem	198	83	99	32.633	15.129	836	120.945
5	Tigecycline	295	118	147.5	42.363	19.172	1,278	174.104
6	Linezolid	180	74	90	29.222	12.871	748	106.827
7	Gentamicin	208	87	98	33.352	15.494	845	127.634
8	Daptomycin	616	273	308	110.913	53.352	2,634	399.677
9	Chloramphenicol	106	47	53	18.998	9.362	456	69.076
10	Tedizolid	174	73	87	29.197	13.046	726	105.544
11	Doxycycline	195	85	97.5	32.972	15.916	852	125.739
12	Cefotaxime	224	78	93	30.783	14.239	784	113.530
13	Imipenem	123	51	61.5	20.213	9.485	516	74.119
14	Meropenem	175	71	87.5	26.673	12.146	742	103.834
15	Vancomycin	662	275	331	105.306	47.656	2,816	401.843
16	Piperacillin	239	98	119.5	37.336	16.973	1,018	143.200

### 2.4 Regression models

Quantifying the relation between any chemical compound’s properties and corresponding TIs is an essential element of QSPR investigation (Shanmukha et al., 2020). Regression analysis and modeling are used as tools in QSPR investigations to accurately predict or estimate the properties of any compound using TIs (Gnanaraj et al., 2023; Shabbir, 2022). A statistical method called regression analysis is used to simulate a dependent variable and several independent variables jointly to evaluate the association between them (Yu et al., 2024). It is widely used in various fields of science and engineering to comprehend how distinct factors modulate the dependent variable and generate predictions. The fundamental concept is to find the line or curve that best captures the association between the variables. The dependent variable, or the attribute that is being investigated to explain or predict, is a response variable. Independent variables, often referred to as predictor variables or explanatory factors, are anticipated to have an impact on the dependent variable. There are various regression models, namely, linear regression, multi-linear regression, logarithmic regression, and curvilinear regression to mention a few. A QSPR model can be built when the desired correlation coefficient (r) $r=\frac{\sum_{i=1}^{N}\left(y_{A_{i}}-{\hat{y}}_{A}\right)\left(y_{P_{i}}-{\hat{y}}_{P}\right)}{\sqrt{\sum_{i=1}^{N}{\left(y_{A_{i}}-{\hat{y}}_{A}\right)}^{2}}\sqrt{\sum_{i=1}^{N}{\left(y_{P_{i}}-{\hat{y}}_{P}\right)}^{2}}}\geq 0.6,$ where ${\;y}_{A_{i}}$ is the physicochemical property of the *i*th compound, ${\hat{y}}_{A}$ is their mean, $y_{P_{i}}$ is the corresponding TI value of the *i*th compound, ${\hat{y}}_{P}$ is the mean of each TI under study, and N is the number of compounds under observation. As per the guidelines set by the International Academy of Mathematical Chemistry, a best quality predictive QSPR model should have the following:

a. $R^{2}$ > 0.8, where $R^{2}=1-\frac{\sum_{i=1}^{N}{\left(y_{A_{i}}-y_{P_{i}}\right)}^{2}}{\sum_{i=1}^{N}{\left(y_{A_{i}}-{\hat{y}}_{A}\right)}^{2}}.$

b. Lowest $RMSE=\sqrt{\frac{1}{N}\sum_{i=1}^{N}{\left(y_{A_{i}}-y_{P_{i}}\right)}^{2}}$ (Adnan Mr et al., 2022; Costa et al., 2021).

There is no universal cutoff (unlike R^2^ values) because the RMSE depends on the scale of the data. Research workers often judge RMSE <10% of the mean value of the physicochemical properties as excellent predictive accuracy and 10%–20% of the mean as acceptable for complex biological/chemical data. F-statistics were used to evaluate the quality of the model fit (Kirmani et al., 2021; Zaman et al., 2024). In the regression model, any trait with a p-value larger than 0.05 and a correlation greater than 0.6 was deemed significant (Jabeen et al., 2024). Because correlation values play an important role in the modeling, they are listed in Tables 7–9.

**TABLE 7:** Correlation values of degree-based TIs and NF medications.

S. no	Physiochemical property	IS	MIR	MR	MD^2^	MH	M^2^DR	M^2^D^2^R
1	Molecular weight	0.993	0.988	0.988	0.987	0.987	0.964	0.99
2	Mass	0.993	0.988	0.987	0.986	0.987	0.964	0.991
3	TPSA	0.949	0.947	0.942	0.939	0.948	0.911	0.945
4	Complexity	0.974	0.986	0.986	0.978	0.987	0.965	0.984
5	Boiling point	0.97	0.986	0.984	0.981	0.986	0.966	0.985
6	Enthalpy of vaporization	0.967	0.984	0.981	0.977	0.983	0.959	0.983
7	Flash point	0.97	0.987	0.984	0.981	0.986	0.966	0.984
8	Molar refractivity	0.983	0.991	0.99	0.988	0.99	0.973	0.992
9	Polar surface area	0.93	0.987	0.942	0.939	0.959	0.911	0.945
10	Polarization	0.983	0.991	0.99	0.988	0.99	0.973	0.992
11	Surface tension	0.443	0.415	0.47	0.45	0.438	0.453	0.476
12	Molar volume	0.973	0.986	0.984	0.982	0.986	0.976	0.982

**TABLE 8:** Correlation values of degree-based TIs and NF medications.

S. no	Physiochemical property	M^2^D^2^	M^2^SD^2^	SD^2^	SC	ABC	M_1_	M_2_
1	Molecular weight	0.993	0.977	0.996	0.992	0.99	0.992	0.992
2	Mass	0.993	0.976	0.996	0.991	0.99	0.992	0.991
3	TPSA	0.945	0.935	0.956	0.96	0.945	0.945	0.97
4	Complexity	0.985	0.98	0.985	0.984	0.981	0.976	0.986
5	Boiling point	0.985	0.971	0.961	0.985	0.981	0.972	0.988
6	Enthalpy of vaporization	0.981	0.962	0.98	0.985	0.983	0.968	0.991
7	Flash point	0.985	0.97	0.984	0.985	0.984	0.972	0.988
8	Molar refractivity	0.991	0.979	0.991	0.992	0.957	0.984	0.994
9	Polar surface area	0.955	0.935	0.956	0.945	0.945	0.932	0.956
10	Polarization	0.991	0.979	0.991	0.993	0.984	0.984	0.994
11	Surface tension	0.462	0.5	0.446	0.462	0.35	0.445	0.89
12	Molar volume	0.982	0.971	0.98	0.984	0.981	0.968	0.992

**TABLE 9:** Correlation values of degree-based TIs and NF medications.

S. no	Physiochemical property	SZ	SK	SK_1_	GA	Randic	HZ	SO
1	Molecular weight	0.97	0.983	0.97	0.996	0.998	0.992	0.983
2	Mass	0.97	0.983	0.971	0.996	0.998	0.992	0.983
3	TPSA	0.914	0.933	0.914	0.943	0.952	0.916	0.933
4	Complexity	0.963	0.976	0.963	0.981	0.985	0.964	0.977
5	Boiling point	0.95	0.972	0.95	0.982	0.987	0.952	0.972
6	Enthalpy of vaporization	0.953	0.968	0.958	0.98	0.987	0.969	0.968
7	Flash point	0.95	0.972	0.958	0.981	0.987	0.952	0.972
8	Molar refractivity	0.97	0.983	0.982	0.99	0.993	0.971	0.984
9	Polar surface area	0.914	0.932	0.914	0.943	0.952	0.916	0.933
10	Polarization	0.97	0.984	0.982	0.99	0.982	0.971	0.984
11	Surface tension	0.487	0.493	0.481	0.448	0.067	0.468	0.463
12	Molar volume	0.948	0.968	0.96	0.979	0.987	0.975	0.968

From the above table, it is evident that the relationship between the properties and indices shows a very good correlation. Hence, linear, quadratic, and cubic regression models were generated to enable better prediction.

### 2.5 Linear regression analysis

The linear regression model can confirm the association because good correlation values are obtained. The TIs obtained for the molecular structures of the NF medications were regarded as independent variables, and the physicochemical attributes were designated as dependent variables. The linear regression models are given by the following equations:

$$P=X_{1}\left(TI\right)+Y\;\left(A\right),$$ (1)

where $X_{1}$ and Y are the regression coefficients. Linear regression models were created using a set of 21 TIs for each of the 12 physicochemical properties using Equation 1. Hence, a set of 252 equations was obtained (Supplementary Information). Few of them are enumerated here for better understanding.

$$MW=11.90\left(IS\right)+1.2178\left(A_{1}\right).$$

$$Mass=76.5833\left(MIR\right)+51.5186\left(A_{2}\right).$$

$$FP=0.3980\left(SO\right)+66.494\left(A_{252}\right).$$

### 2.6 Quadratic regression model

Quadratic regression is a strong statistical method for modeling associations that take the form of a parabolic curve between a dependent variable and one or more independent variables. This captures the curving trends and more intricate data patterns by fitting a second-degree polynomial to the data points. By exploring the underlying patterns in their datasets, investigators and analysts can enhance forecast accuracy, gain a deeper understanding of data trends, and make better decisions. The model is defined by the following equation:

$$P=X_{1}\left(TI\right)+X_{2}{\left(TI\right)}^{2}+Y\;\left(B\right),$$

where P represents the property and $X_{1},X_{2},Y$ are the constant regression coefficients. Among the 252 equations, a few are listed in the following equations:

$$MW={0.0240\left(IS\right)}^{2}+8.1879\left(IS\right)+103.048\left(B_{1}\right).$$

$$MW=1.6147{\left(MIR\right)}^{2}+40.268\left(MIR\right)+190.731\left(B_{2}\right).$$

$$FP=0.0001{\left(SO\right)}^{2}-0.0524\left(SO\right)+318.913\left(B_{252}\right).$$

### 2.7 Cubic regression

Cubic regression is a nonlinear statistical approach in which the dependent property (e.g., solubility and logP) is modeled as a cubic function of the molecular descriptors. Unlike linear or quadratic models, cubic regression can capture more complex, non-monotonic relationships between structural features and physicochemical properties, improving the predictive accuracy when higher-order interactions exist. The model is defined by the following equation:

$$P=X_{1}\left(TI\right)+X_{2}{\left(TI\right)}^{2}+X_{3\;}{\left(TI\right)}^{3}+Y\;\left(C\right),$$

where P represents the property and $X_{1},X_{2},X_{3\;},Y$ are the constant regression coefficients.

$$MW=-0.0003{\left(IS\right)}^{3}+{0.0869\left(IS\right)}^{2}+4.5057\left(IS\right)+165.1323\left(C_{1}\right).$$

$$MW=-0.00773{\left(MIR\right)}^{3}+1.8563{\left(MIR\right)}^{2}+38.2208\left(MIR\right)+195.5547\left(C_{2}\right).$$

$$\text{FP}=5.36E-08{\left(SO\right)}^{3}+-9.3E-05{\left(S0\right)}^{2}+0.2554\left(SO\right)+206.6436\left(C_{252}\right).$$

### 2.8 Multi-attribute decision analysis of drugs

Drug design requires optimization across multiple, often contradictory, molecular features, such as ensuring solubility without sacrificing permeability, achieving a strong binding affinity with minimal toxicity, and balancing molecular weight with acceptable bioavailability. Assessing these criteria in isolation frequently creates trade-offs that complicate the lead selection process. Degree-based TIs provide a compact mathematical representation of molecular connectivity and demonstrate strong correlations with physicochemical descriptors, including polarity, lipophilicity, and mass. When incorporated into QSPR models, TIs allow predictive evaluation of pharmacokinetic and pharmacodynamic behavior. MCDM methods, such as TOPSIS and MOORA, integrate these descriptors into composite rankings, facilitating rational prioritization of NF antibiotics (Hezer et al., 2021; Feng et al., 2022).

### 2.9 TOPSIS ranking

**FIGURE 3:**
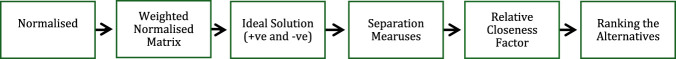
Workflow process of the TOPSIS method.

The modified TOPSIS approach is based on identifying ideal and anti-ideal solutions, which are then used to calculate the distances among the different options and the ideal solution. This approach addresses qualitative and quantitative factors, and ranks the options based on their proximity to the ideal solution (Chakraborty, 2022; Madanchian and Taherdoost, 2023). The various steps involved in the process are presented in Figure 3.

The generic MADM problem aims to access and rank alternatives based on certain attributes ( X ). The alternatives represent the available options for the decision-maker, which require ranking. The set of attributes (A_i_) represents the factors influencing the decision-makers’ choice while ranking the alternatives. Performance ratings for each alternative against each attribute are represented in the form of a decision matrix defined by X.

$$X=\sum_{n=1}^{k}\sum_{m=1}^{i}x_{mn}.$$

To represent the relative significance of the attribute’s weights, $W=\sum_{n=1}^{k}w_{n}=1$ .

The MADM problem is represented by $ℵ=\left\{X,W\;\right\}$ .

This problem is solved using the modified TOPSIS method. The process is enumerated in the following stages.

Stage 1: calculate the normalized performance ratings $\beta_{mn}=\frac{x_{mn}}{\sqrt{\sum_{m=1}^{i}{x_{mn}}^{2}}}$ .

The normalized ratings $\beta_{mn}$ can be represented as a matrix $\theta =\sum_{n=1}^{k}\sum_{m=1}^{i}\left[\theta_{mn}\right]$ .

Stage 2: integrate weight with the ratings. The weight and ratings are combined to form the weighted normalized decision matrix $V=\vartheta_{mn}=\sum_{n=1}^{k}\sum_{m=1}^{i}\left[\vartheta_{mn}\right]$ , where $\vartheta_{mn}=$ $w_{n}\times$ $\beta_{mn}\left(m=1,2\ldots i,n=1,2\ldots .j\right)$ .
Stage 3: identify the positive and negative ideal solutions $\alpha^{+}$ and $\alpha^{-}$ , calculated from $\alpha^{+}=[$ $\left.{\vartheta_{1}}^{+},{\vartheta_{2}}^{+},\ldots \ldots .{\vartheta_{m}}^{+}\right]$ , $\alpha^{-}=[$ $\left.{\vartheta_{1}}^{-},{\vartheta_{2}}^{-},\ldots \ldots .{\vartheta_{m}}^{-}\right]$ , where ${\vartheta_{m}}^{+}=\max\vartheta_{mn}$ and ${\vartheta_{m}}^{-}=\min\vartheta_{mn}$ .
Stage 4: obtain the weighted Euclidean distance.

$${\gamma_{m}}^{+}=\sqrt{\sum_{n=1}^{j}w_{n}\;{\left(\vartheta_{mn}-{\vartheta_{m}}^{+}\right)}^{2}\;}\text{ and }{\gamma_{m}}^{-}=\sqrt{\sum_{n=1}^{j}w_{n}\;{\left(\vartheta_{mn}-{\vartheta_{m}}^{-}\right)}^{2}\;}.$$

Stage 5: obtain the overall performance score

$$\rho_{m}=\frac{{\gamma_{m}}^{-}}{{\gamma_{m}}^{+}+{\gamma_{m}}^{-}}.$$

The performance score $\rho_{m}$ is used to rank the alternatives. A higher score indicates better alternative performance (Chakraborty, 2022; Madanchian and Taherdoost, 2023).

### 2.10 MOORA ranking

MOORA, developed by Brauers and Zavadskas, is a widely used MCDM approach. It is computationally efficient, conceptually simple, and particularly effective in addressing scenarios that involve multiple conflicting criteria. Its ability to integrate diverse molecular descriptors makes it highly suitable for drug design, where trade-offs among solubility, bioavailability, and toxicity are prevalent. MOORA is robust against normalization bias, ensuring consistent and reliable results when applied to drug-like property datasets. These features make it a valuable tool for QSPR studies and rational compound prioritization. The performance index $y_{m}$ , which is required for MOORA ranking, is calculated from the normalized matrix and assigned weights (attained from steps 1 and 2) using the following equation:

$$y_{m}=\sum_{n=1}^{g}w_{n}{x_{mn}}^{*}-\sum_{j=g+1}^{l}w_{n}{x_{mn}}^{*}.$$

Here, $y_{m}$ represents the performance index of the alternative m , g represents the number of beneficial criteria, and l represents the total number of criteria. The alternatives are ranked based on the descending order of $y_{m}.$

## 3 Results

A substantial set of 252 equations was created for each of the three regression models. This set was generated using 21 degree-based indices related to each of the 12 studied physicochemical properties. Finally, 756 equations were generated for the models, and their corresponding intercepts and constants were calculated.

Among all the models, the best predictive models were chosen using the factors essential for comprehension, as mentioned in Section 2.4.

The models exhibited strong correlations and reproducibility. Hence, the analysis indicates strong associations between TIs and NF drugs. This firmly supports the validity of our models. Consequently, the p-values for each parameter in the regression models were investigated. The best-fit regression models based on the aforementioned statistical parameters for the linear and quadratic QSPR models filtered from the large dataset are presented, and the rest of the regression equations are given in the Supplementary Information. Best-fit regression plots were calculated using Microsoft Excel software. The best predictions of the linear regression models and their statistical parameters are listed in Table 10.

**TABLE 10:** Statistical parameters of the linear regression model.

S. no	Property	TI	R^2^	F	P-value	A	B
1	MW	Randic	0.985	912.817	3.78 ${\times 10}^{-14}$	19.450	30.618
2	Mass	Randic	0.985	915.457	3.71 ${\times 10}^{-14}$	19.371	30.594
3	TPSA	M_2_	0.916	151.910	6.63 ${\times 10}^{-9}$	3.892	26.664
4	Complexity	MH	0.974	533.581	1.51 ${\times 10}^{-12}$	−94.130	118.895
5	MR	Randic	0.988	1150.829	7.63 ${\times 10}^{-15}$	4.079	7.481
6	PSA	MH	0.921	75.295	0.001205	19.657	37.952
7	Polarization	Randic	0.988	1152.178	7.57 ${\times 10}^{-15}$	1.608	2.966
8	MV	M_2_	0.984	851.032	6.1361 ${\times 10}^{-14}$	8.663	42.450
9	BP	M_2_	0.976	411.362	1.87 ${\times 10}^{-9}$	240.582	72.948
10	EV	M_2_	0.980666	507.2222	6.7 ${\times 10}^{-10}$	26.885	13.361
11	FP	M_2_	0.976274	411.4715	1.87 ${\times 10}^{-9}$	99.267	44.121

Table 11 displays the visualization of the predicted and actual values (provided in Supplementary Information) from the best linear regression model (the scripting notations in the equations were replaced with x and y in the figures for convenient readability) using Python.

**TABLE 11:** Best-fit linear regression of the predicted and actual values for NF medications.

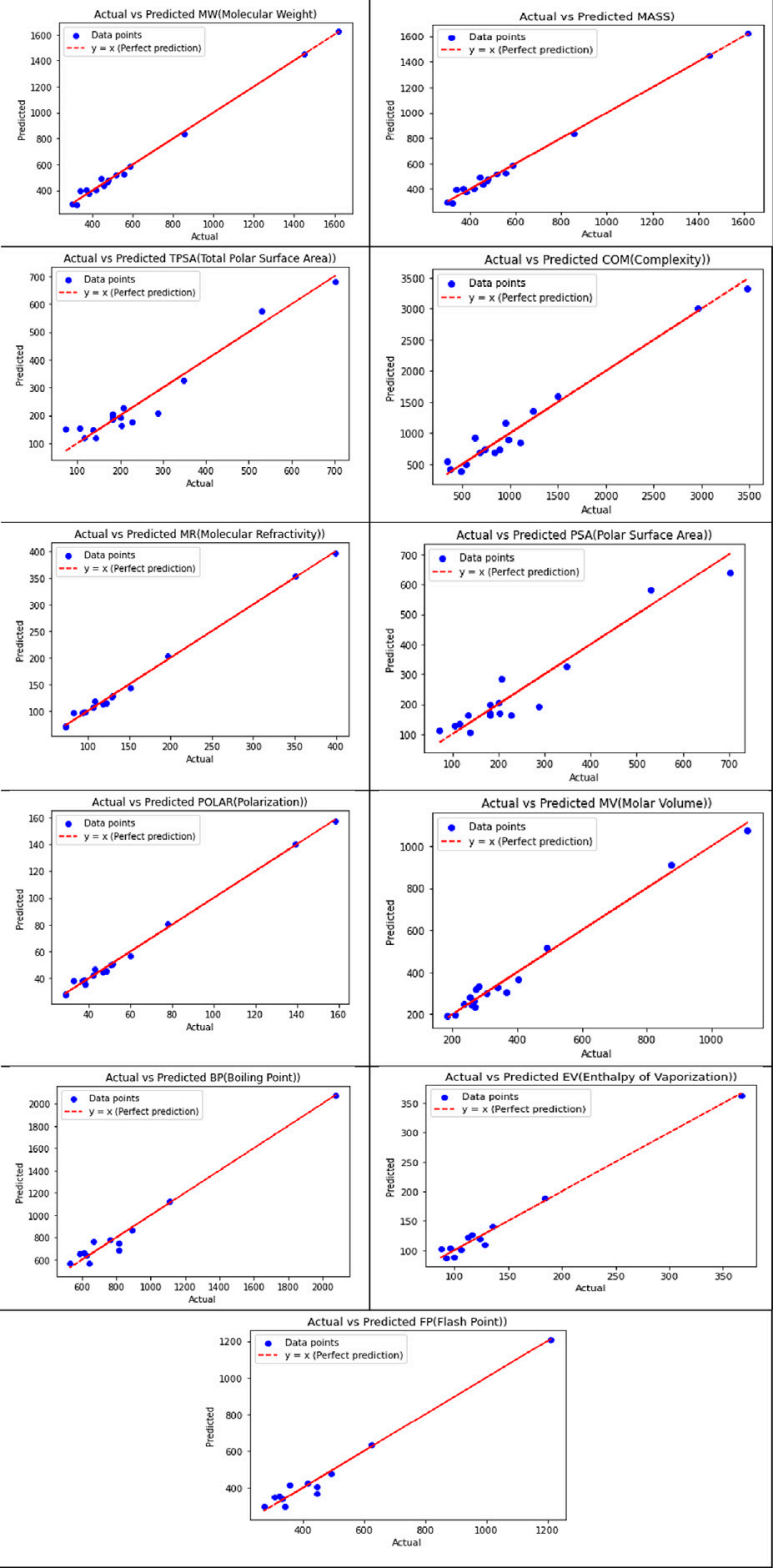

Of the 252 equations, six properties yielded the best-fit quadratic regression models, which are summarized in Table 12.

**TABLE 12:** Statistical parameters of the quadratic regression model.

S. no	Property	TI	R^2^	F	P-value	A	B	C
1	MW	SD^2^	0.986143	462.5707	9.10 ${\times 10}^{-4}$	161.3338	3.047502	0.008157
2	Mass	SD^2^	0.986144	462.6065	8.86 ${\times 10}^{-4}$	160.7115	3.052868	0.008126
3	MR	SD^2^	0.98959	617.9065	5.30 ${\times 10}^{-4}$	40.00031	0.72244	0.002062
4	Polarization	SD^2^	0.989639	620.8425	2.13 ${\times 10}^{-4}$	15.87175	0.28606	0.000819
5	MV	MIR	0.982622	367.5381	2.06 ${\times 10}^{-1}$	139.6502	23.25806	1.202926
6	EV	SZ	0.979415	214.1038	7.67 ${\times 10}^{-4}$	118.4714	−0.28668	0.001205

Table 13 illustrates the predicted and actual values (provided in supplementary information) from the best quadratic regression model derived from this analysis using Python.

**TABLE 13:** Best-fit quadratic regression of the predicted and actual values for NF medications.

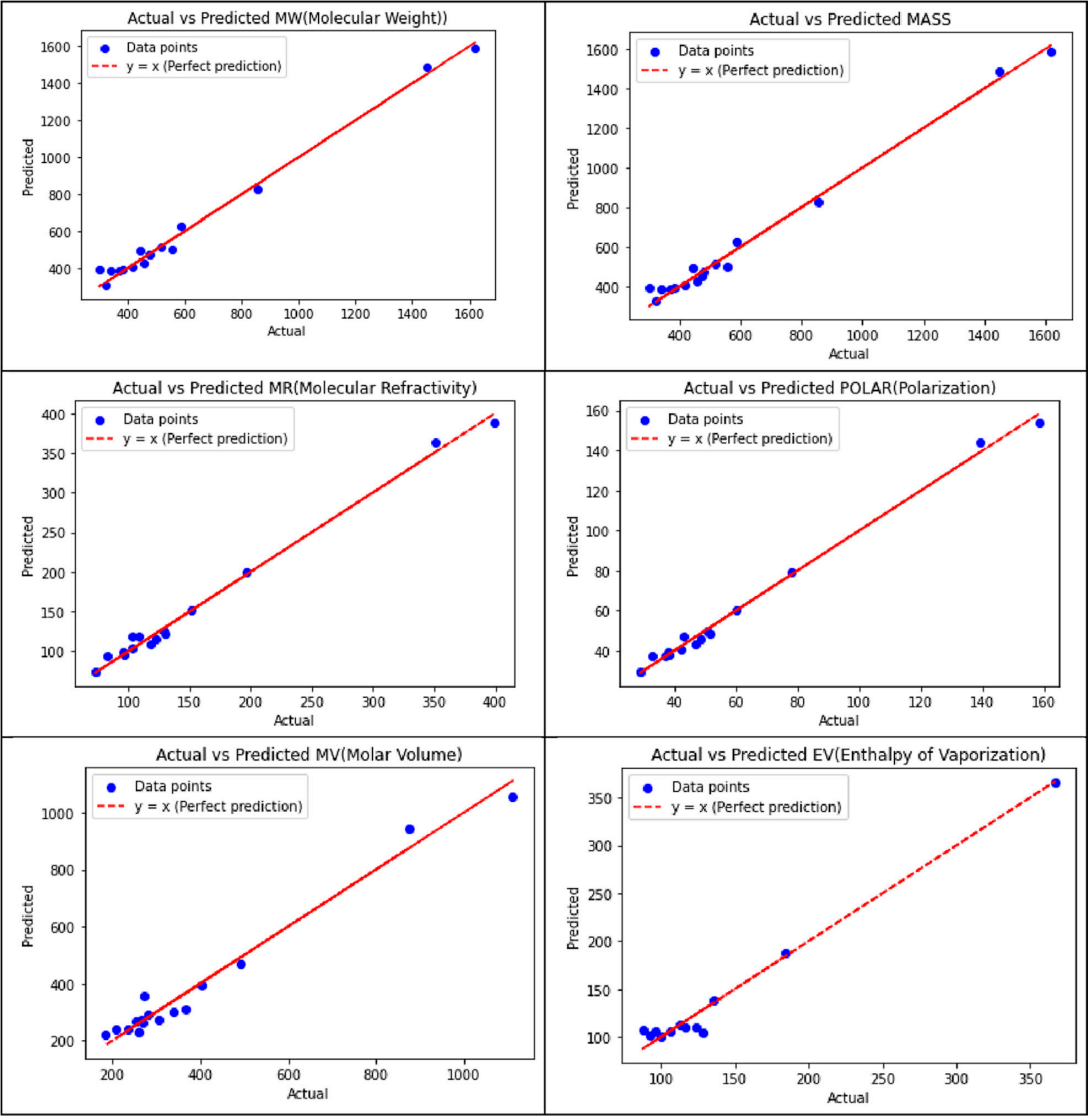

Of the 252 equations, four properties yielded the best-fit cubic regression models of the predicted and actual values, as summarized in Table 14 using python.

**TABLE 14:** Statistical parameters of the cubic regression model.

S. no	Property	TI	R^2^	F	P-value	A	B	C	D
1	Complexity	SD^2^	0.9856	274.163	0.0478	−845.329	31.4154	−0.1636	0.0004
2	MR	SD^2^	0.9961	1019.867	0.04614	−20.9484	2.1873	−0.0086	2.15 $\times 10^{-5}$
3	Polarization	SD^2^	0.9961	1025.139	0.0461	−8.23937	0.8656	−0.0034	8.49 $\times 10^{-6}$
4	MV	SD^2^	0.9831	232.009	0.0482	−127.338	8.3311	−0.0476	0.000118

Table 15 illustrates the predicted and actual values from the best cubic regression model derived from this analysis using Python.

**TABLE 15:** Best-fit cubic regression of the predicted and actual values for NF medications.

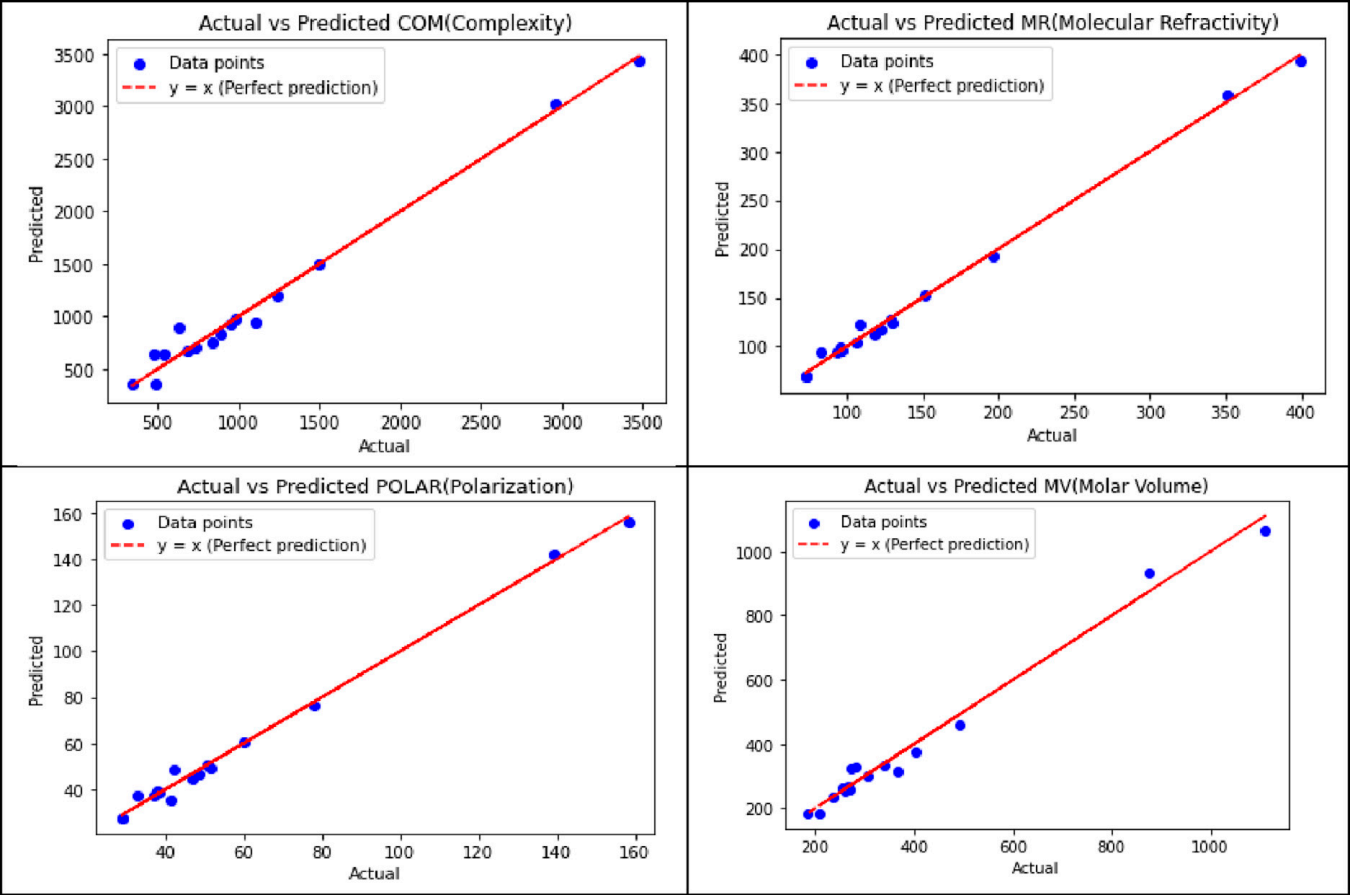

### 3.1 TOPSIS ranking of antibiotics for NF

Among the 21 valency-based indices used in this study, six indices produced the best-fit regression models. These TI values were considered for ranking the NF antibiotics. The attributes and alternates used for ranking are displayed in Table 16.

**TABLE 16:** TIs (attributes) utilized for ranking of NF medications (alternates).

S. no	Drug compound	MIR	MH	SD^2^	M_2_	SZ	Randic
1	Ceftriaxone	5.62	8.333	91.667	7.889	231	17.116
2	Aminoglycoside 66-40C	9.391	13.667	149.5	11.75	394	26.299
3	Cephalosporin C	4.842	7.333	71.333	6.222	163	13.129
4	Ertapenem	4.842	7.333	81.333	7.056	198	15.129
5	Tigecycline	8.419	12.667	116.917	8.681	295	19.172
6	Linezolid	3.833	5.667	64.333	6.278	148	12.956
7	Gentamicin	5.093	7.5	77.75	6.764	200	14.494
8	Daptomycin	19.883	29.5	274.833	25.278	586	52.427
9	Chloramphenicol	3.737	5.667	49.667	4.611	106	9.362
10	Tedizolid	3.767	5.333	67.333	5.889	174	13.046
11	Doxycycline	5.641	8.667	87.5	6.778	237	14.867
12	Cefotaxime	3.251	5.167	49.833	4.722	111	9.415
13	Imipenem	2.795	4.333	49.667	4.556	123	9.485
14	Meropenem	4.582	7	69	5.583	175	12.146
15	Vancomycin	17.606	25.833	254.5	21.028	626	46.203
16	Piperacillin	5.963	8.833	94.75	7.847	236	17.048

The weights in this modified TOPSIS model indicate the proportion of a drug structure that must be considered. The drug structure has a wide range of influences on the chemical and physical characteristics. Hence, the weights were allocated according to the analytical hierarchy process. The relative importance of each pair of items is acquired from the results of the best-fit criteria. The weight allocation satisfying the condition $\sum_{n=1}^{k}w_{n}=1$ is presented in Table 17.

**TABLE 17:** Weights assigned for TIs.

Index	MIR	MH	SD^2^	M_2_	SZ	Randic
Weight	0.08	0.08	0.18	0.48	0.04	0.14

The weighted normalized matrix calculated according to the procedure explained in stage 2 is presented in Table 18.

**TABLE 18:** Normalized matrix of NF medications.

S. no	Drug compound	MIR	MH	SD^2^	M_2_	SZ	Randic
1	Ceftriaxone	0.16805	0.16807	0.18713	0.18761	0.19735	0.00347
2	Aminoglycoside 66-40C	0.28081	0.27565	0.30519	0.27943	0.33661	0.07981
3	Cephalosporin C	0.14479	0.1479	0.14562	0.14797	0.13926	0.00266
4	Ertapenem	0.14479	0.1479	0.16603	0.1678	0.16916	0.00306
5	Tigecycline	0.25175	0.25548	0.23868	0.20645	0.25203	0.00388
6	Linezolid	0.25175	0.25548	0.23868	0.20645	0.12644	0.00262
7	Gentamicin	0.15229	0.15127	0.15872	0.16086	0.17087	0.00294
8	Daptomycin	0.59455	0.59498	0.56105	0.60115	0.50065	0.01062
9	Chloramphenicol	0.11174	0.1143	0.10139	0.10966	0.09056	0.0019
10	Tedizolid	0.11264	0.10756	0.13745	0.14005	0.14866	0.00264
11	Doxycycline	0.16868	0.1748	0.17862	0.16119	0.20248	0.00301
12	Cefotaxime	0.09721	0.10421	0.10173	0.1123	0.09483	0.00191
13	Imipenem	0.08358	0.08739	0.10139	0.10835	0.10509	0.00192
14	Meropenem	0.13701	0.14118	0.14086	0.13277	0.14951	0.00246
15	Vancomycin	0.52646	0.52102	0.51954	0.50008	0.53482	0.00936
16	Piperacillin	0.17831	0.17815	0.19342	0.18661	0.20163	0.00345

The positive and negative ideal solutions calculated in stage 3 are listed in Table 19.

**TABLE 19:** Positive and negative ideal solutions of NF medications.

Ideal solutions	MIR	MH	SD^2^	M_2_	SZ	Randic
V+	0.04756	0.0476	0.10099	0.28855	0.02139	0.01117
V-	0.00669	0.00699	0.01825	0.05201	0.00362	0.00027

The weighted normalized matrix of the attributes and alternates of NF antibiotics is presented in Table 20.

**TABLE 20:** Weighted normalized matrix of NF antibiotics.

S. no	Drug compound	MIR	MH	SD^2^	M_2_	SZ	Randic
1	Ceftriaxone	0.01344	0.01345	0.03368	0.09005	0.00789	0.00049
2	Aminoglycoside 66-40C	0.02246	0.02205	0.05493	0.13413	0.01346	0.01117
3	Cephalosporin C	0.01158	0.01183	0.02621	0.07102	0.00557	0.00037
4	Ertapenem	0.01158	0.01183	0.02989	0.08055	0.00677	0.00043
5	Tigecycline	0.02014	0.02044	0.04296	0.09909	0.01008	0.00054
6	Linezolid	0.02014	0.02044	0.04296	0.09909	0.00506	0.00037
7	Gentamicin	0.01218	0.0121	0.02857	0.07721	0.00683	0.00041
8	Daptomycin	0.04756	0.0476	0.10099	0.28855	0.02003	0.00149
9	Chloramphenicol	0.00894	0.00914	0.01825	0.05264	0.00362	0.00027
10	Tedizolid	0.00901	0.0086	0.02474	0.06722	0.00595	0.00037
11	Doxycycline	0.01349	0.01398	0.03215	0.07737	0.0081	0.00042
12	Cefotaxime	0.00778	0.00834	0.01831	0.0539	0.00379	0.00027
13	Imipenem	0.00669	0.00699	0.01825	0.05201	0.0042	0.00027
14	Meropenem	0.01096	0.01129	0.02535	0.06373	0.00598	0.00034
15	Vancomycin	0.04212	0.04168	0.09352	0.24004	0.02139	0.00131
16	Piperacillin	0.01426	0.01425	0.03482	0.08957	0.00807	0.00048

The weighted Euclidean distance and performance scores of the NF antibiotics and their corresponding rankings calculated as the final stage of TOPSIS and MOORA are listed in Tables 21, 22, respectively.

**TABLE 21:** Ranking of NF antibiotics by the TOPSIS method.

S. no	Drug compound	Si+	Si-	Pi	Rank
1	Ceftriaxone	0.21577	0.05074	0.19037	7
2	Aminoglycoside 66-40C	0.16527	0.09592	0.36725	3
3	Cephalosporin C	0.23633	0.03756	0.13713	11
4	Ertapenem	0.22633	0.04317	0.16018	8
5	Tigecycline	0.20246	0.06222	0.23508	5
6	Linezolid	0.20281	0.06449	0.24127	4
7	Gentamicin	0.22967	0.04075	0.15068	10
8	Daptomycin	0.00978	0.25764	0.96343	1
9	Chloramphenicol	0.25672	0.03273	0.11308	14
10	Tedizolid	0.24117	0.0346	0.12546	12
11	Doxycycline	0.22785	0.04149	0.15404	9
12	Cefotaxime	0.25583	0.03251	0.11274	15
13	Imipenem	0.25794	0.032	0.11036	16
14	Meropenem	0.24347	0.03373	0.12169	13
15	Vancomycin	0.05071	0.20904	0.80478	2
16	Piperacillin	0.2156	0.05087	0.19089	6

**TABLE 22:** Ranking of NF antibiotics using MOORA.

S. no	Drug compound	Mean (normalized)	Std dev (normalized)	Performance index	Rank
1	Ceftriaxone	0.1830	0.0122	0.1851	6
2	Aminoglycoside 66-40C	0.2950	0.0231	0.2879	3
3	Cephalosporin C	0.1452	0.0032	0.1466	10
4	Ertapenem	0.1606	0.0111	0.1641	8
5	Tigecycline	0.2362	0.0214	0.2225	4
6	Linezolid	0.1300	0.0146	0.1388	11
7	Gentamicin	0.1592	0.0071	0.1594	9
8	Daptomycin	0.5724	0.0379	0.5862	1
9	Chloramphenicol	0.1053	0.0087	0.1071	14
10	Tedizolid	0.1319	0.0174	0.1358	13
11	Doxycycline	0.1751	0.0148	0.1682	7
12	Cefotaxime	0.1025	0.0062	0.1068	15
13	Imipenem	0.0985	0.0104	0.1029	16
14	Meropenem	0.1394	0.0060	0.1362	12
15	Vancomycin	0.5192	0.0119	0.5106	2
16	Piperacillin	0.1879	0.0091	0.1875	5

The rank correlation coefficients between TOPSIS and MOORA rankings of NF antibiotics by Spearman’s rank correlation (ρ) = 0.912, which is very high and statistically significant, showing a strong positive correlation between the two ranking methods. This confirms that although minor differences exist in mid-tier drugs, TOPSIS and MOORA are largely consistent in identifying the top and bottom performers.

## 4 Discussion

The molecular modeling framework utilized in this study uses complex simulations and regression analyses to predict the antibiotic behavior. Using 21 TIs correlated with 12 physicochemical properties, 504 equations were generated, of which 17 best-fit models were proven to be reliable for pharmacological repurposing. These results demonstrate that six indices—M2, Randić, SD2, MIR, SZ, and MH—consistently provided the strongest predictive power, with M2 emerging as the most effective index across multiple models. The weighted integration of these indices within a modified TOPSIS framework enabled the accurate ranking of NF antibiotics, supporting precise forecasting of drug properties. This ranking approach can guide pharmaceutical companies in prioritizing promising candidates for NF therapy, thereby reducing the costs and risks of clinical trials. Overall, this study establishes a robust quantitative methodology for linking molecular configurations to physicochemical properties, thus offering systematic applications in drug design and repurposing. A comparative analysis of the TOPSIS and MOORA methods revealed a strong consistency in identifying the best- and worst-performing antibiotics for NF.

**FIGURE 4:**
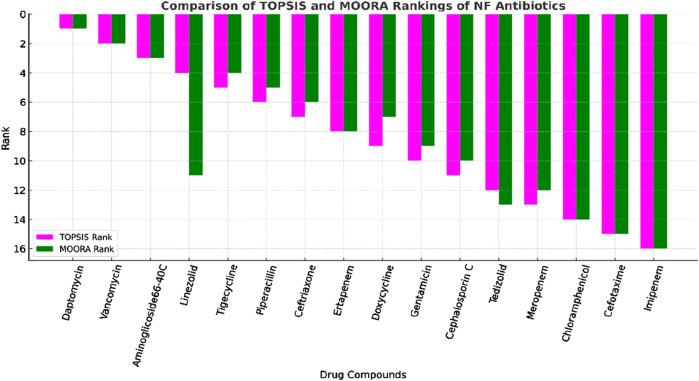
Ranking analysis of NF antibiotics based on TOPSIS and MOORA.

The comparative ranking is visualized in Figure 4.

Daptomycin ranked first in both methods, confirming its superior performance, whereas vancomycin consistently ranked second, reflecting its strong structural and physicochemical profile. Aminoglycoside 66-40C was also ranked third by both, supported by MIR, MH, and S $D^{2}$ . Minor divergences appeared in mid-tier drugs, such as linezolid, which was ranked fourth by TOPSIS but eleventh by MOORA. Tigecycline, piperacillin, and ceftriaxone showed close agreement, and ertapenem matched exactly at the eighth position. Doxycycline and gentamicin were similarly placed mid-range. Lower-tier antibiotics, including cephalosporin C, tedizolid, and meropenem, were consistent across both methods, whereas chloramphenicol, cefotaxime, and imipenem ranked the lowest. This strong alignment highlights the robustness of MCDM approaches for drug prioritization. Overall, the predictive QSPR models and rankings demonstrated reliability, providing a quantitative foundation for NF drug repurposing and wider applications in drug design, materials science, environmental chemistry, and cheminformatics.

## 5 Limitations and future work

Although the present analysis highlights the predictive strength of six degree-based topological indices (TIs) for modeling the physicochemical properties of antibiotics used to treat NF, certain limitations should be acknowledged. Out of the 21 indices considered, only a subset yielded statistically significant correlations, suggesting that several descriptors may lack discriminative ability for the dataset. This selective outcome also indicates the potential risk of overfitting, especially because the regression models were derived from a relatively small number of drugs. Furthermore, the scope of the study was limited to degree-based indices, excluding distance-, entropy-, and spectral-based descriptors that could capture the complementary structural features of the networks. The QSPR models were statistically validated but not tested against independent external datasets, which may restrict the generalizability of these findings. Similarly, the MCDM-based ranking depends on the selected criteria and weighting schemes, which introduce subjectivity into prioritization. Future studies should expand the dataset to include a broader range of antibiotics, integrate hybrid or machine learning-based models, and employ external validation to strengthen the predictive reliability. Incorporating diverse topological descriptors may also enhance the robustness of the drug-ranking frameworks.

## 6 Conclusion

In this study, we demonstrate the potential of degree-based TIs combined with regression and multi-criteria decision-making techniques to predict and rank the physicochemical properties of antibiotics used to treat NF. Among the 21 indices analyzed, six (M_2_, Randić, SD^2^, MIR, SZ, and MH) consistently produced the best-fit QSPR models, with M_2_ being the strongest predictor. The weighted integration of these indices within a modified TOPSIS framework, supported by MOORA validation, provided robust and consistent antibiotic rankings. These findings highlight a systematic, quantitative approach for drug repurposing and prioritization, offering valuable insights into rational NF therapy development.

## Data Availability

The original contributions presented in the study are included in the article/Supplementary Material, further inquiries can be directed to the corresponding author.
