# Supplementary material for: Quantitative structure–property relationship modeling and ranking of necrotizing fasciitis drugs via degree-based topological indices
Source: Front Chem. 2025 Oct 28;13:1668093. doi: 10.3389/fchem.2025.1668093 (PMC12602421; doi:10.3389/fchem.2025.1668093)
Supplement: Supplementary file 1 [file DataSheet1.docx]

Quantitative Structure–Property Relationship Modeling and Ranking of Necrotizing Fasciitis Drugs via Degree-Based Topological Indices

Joy Prisca A^1^ , B. Jaganathan^1*^

^1^Department of Mathematics, School of Advanced Sciences, Vellore Institute of Technology, Chennai, India

email id: [joyprisca.a2020@vitstudent.ac.in](mailto:joyprisca.a2020@vitstudent.ac.in)

**Corresponding author: jaganathan.b@vit.ac.in**

**Linear Regression Equations of NF Anti-Biotics**

Molecular Weight:

$$MW=11.90 \left( IS \right)+1.2178$$

$$MW=76.6435\left( MIR \right)+51.6145$$

$$MW=38.1189 \left( MR \right)+60.7626$$

$$MW=12.9530 \left( MDD \right)+65.2268$$

$$MW=52.1328 \left( MHD \right)+47.6857$$

$$MW=9.9154 \left( M^{2}DR \right)+74.2267$$

$$MW=10.4081 \left( M^{2}D^{2}R \right)+8.7497$$

$$MW=7.6411 \left( M^{2}D^{2} \right)+15.2088$$

$$MW=3.546164 \left( M^{2}{SD}^{2} \right)+26.0339$$

$$MW=5.5782 \left( SDD \right)+1.399$$

$$MW=29.5497\left( SCD \right)+2.8268$$

$$MW=18.6413(ABC)+1.7472$$

$$MW=2.7730\left( M_{1} \right)-5.5491$$

$$MW=64.8191 \left( M_{2} \right)+10.61$$

$$MW=2.3671 \left( SZ \right)-20.9435$$

$$MW=5.546 \left( SK \right)-5.5491$$

$$MW=4.7027 \left( {SK}_{1} \right)-9.4679$$

$$MW=14.1520 \left( GA \right)-0.4192$$

$$MW=30.3255 \left( Randic \right)+5.6221$$

$$MW=3.7882\left( HZ \right)-4.6762$$

$$MW=0.55 \left( SO \right)-7.2367$$

Mass :

$$Mass=11.8906 \left( IS \right)+1.1653$$

$$Mass=76.5833 \left( MIR \right)+51.5186$$

$$Mass=38.0889 \left( MR \right)+60.6593$$

$$Mass=12.9492 \left( MDD \right)+65.119$$

$$Mass=52.0918 \left( MHD \right)+47.17582$$

$$Mass=9.9074\left( M^{2}DR \right)+74.1222$$

$$Mass=10.4 \left( M^{2}D^{2}R \right)+8.6846$$

$$Mass=7.6351\left( M^{2}D^{2} \right)+15.1379$$

$$Mass=3.5434 \left( M^{2}{SD}^{2} \right)+25.95$$

$$Mass=5.5739\left( SDD \right)+1.3391$$

$$Mass=29.5268\left( SCD \right)+2.7638$$

$$Mass=18.6269(ABC)+1.6865$$

$$Mass=2.7708\left( M_{1} \right)-5.5989$$

$$Mass=64.7695\left( M_{2} \right)+10.535$$

$$Mass=2.3652 \left( SZ \right)-20.9754$$

$$Mass=5.5415 \left( SK \right)-5.5989$$

$$Mass=4.6988 \left( {SK}_{1} \right)-9.5088$$

$$Mass=14.141 \left( GA \right)-0.4773$$

$$Mass=30.3021 \left( Randic \right)+5.5548$$

$$Mass=3.7852 \left( HZ \right)-4.7271$$

$$Mass=0.5496 \left( SO \right)-7.2807$$

Total Polar Surface Area:

$$TPSA=4.8160 \left( IS \right)-2.7354$$

$$TPSA=31.3337 \left( MIR \right)+15.4127$$

$$TPSA=15.5081 \left( MR \right)+20.2537$$

$$TPSA=5.2564 \left( MDD \right)+22.588$$

$$TPSA=21.3622 \left( MHD \right)+13.1178$$

$$TPSA=3.9584 \left( M^{2}DR \right)+29.6877$$

$$TPSA=4.2302 \left( M^{2}D^{2}R \right)-0.7012$$

$$TPSA=3.1295 \left( M^{2}D^{2} \right)+9.3541$$

$$TPSA=2.26653 \left( M^{2}{SD}^{2} \right)-3.6205$$

$$TPSA=2.2881 \left( SDD \right)+2.8023$$

$$TPSA=12.0393 \left( SCD \right)-3.700$$

$$TPSA=7.5762 (ABC)-3.5387$$

$$TPSA=1.1181\left( M_{1} \right)-4.5642$$

$$TPSA=26.6896 \left( M_{2} \right)-3.0718$$

$$TPSA=0.9517 \left( SZ \right)-10.0585$$

$$TPSA=2.2361 \left( SK \right)-4.5642$$

$$TPSA=1.8815 \left( {SK}_{1} \right)-4.2514$$

$$TPSA=5.744 \left( GA \right)-4.1079$$

$$TPSA=12.4009 \left( Randic \right)-3.4508$$

$$TPSA=1.5278 \left( HZ \right)-4.2775$$

$$TPSA=0.2201 \left( SO \right)+0.0232$$

Complexity:

$$\mathrm{Complexity}=26.5784 \left( IS \right)-216.854$$

$$\mathrm{Complexity}=172.516 \left( MIR \right)-113.797$$

$$\mathrm{Complexity}=85.5104 \left( MR \right)-89.1089$$

$$\mathrm{Complexity}=29.0126\left( MDD \right)-77.2681$$

$$\mathrm{Complexity}=117.5554\left( MHD \right)-125.804$$

$$\mathrm{Complexity}=22.3794\left( M^{2}DR \right)-66.116$$

$$\mathrm{Complexity}=23.3075 \left( M^{2}D^{2}R \right)-203.487$$

$$\mathrm{Complexity}=17.1463 \left( M^{2}D^{2} \right)-191.707$$

$$\mathrm{Complexity}=7.9941\left( M^{2}{SD}^{2} \right)-173.338$$

$$\mathrm{Complexity}=12.5016\left( SDD \right)-221.014$$

$$\mathrm{Complexity}=66.1080 \left( SCD \right)-215.456$$

$$\mathrm{Complexity}=41.7172\left( ABC \right)-128.292$$

$$\mathrm{Complexity}=6.1938\left( M_{1} \right)-232.067$$

$$\mathrm{Complexity}=145.4554 \left( M_{2} \right)-202.062$$

$$\mathrm{Complexity}=5.2839 \left( SZ \right)-265.537$$

$$\mathrm{Complexity}=12.3877 \left( SK \right)-232.067$$

$$\mathrm{Complexity}=10.5033 \left( {SK}_{1} \right)-240.7$$

$$\mathrm{Complexity}=31.6252 \left( GA \right)-221.225$$

$$\mathrm{Complexity}=67.9295 \left( Randic \right)-210.879$$

$$\mathrm{Complexity}=8.4645 \left( HZ \right)-230.573$$

$$\mathrm{Complexity}=1.2291 \left( SO \right)-236.346$$

Boiling Point:

$$BP=14.0022 \left( IS \right)+206.0485$$

$$BP=87.1358 \left( MIR \right)+266.3463$$

$$BP=43.5372 \left( MR \right)+275.1565$$

$$BP=14.7977 \left( MDD \right)+280.3287$$

$$BP=59.0377 \left( MHD \right)+262.9309$$

$$BP=11.7813 \left( M^{2}DR \right)+274.2844$$

$$BP=11.9625 \left( M^{2}D^{2}R \right)+221.0612$$

$$BP=8.755 \left( M^{2}D^{2} \right)+227.1082$$

$$BP=4.0006 \left( M^{2}{SD}^{2} \right)+242.0118$$

$$BP=6.4031 \left( SDD \right)+213.4248$$

$$BP=33.6102 \left( SCD \right)+221.9815$$

$$BP=21.3847(ABC)+215.8859$$

$$BP=3.2552 \left( M_{1} \right)+197.3248$$

$$BP=71.6828 \left( M_{2} \right)+241.7778$$

$$BP=2.825 \left( SZ \right)+179.5859$$

$$BP=6.5103 \left( SK \right)+197.3248$$

$$BP=5.6488 \left( {SK}_{1} \right)+184.5523$$

$$BP=16.2737 \left( GA \right)+213.8485$$

$$BP=34.1516 \left( Randic \right)+228.9094$$

$$BP=4.4426 \left( HZ \right)+1983646$$

$$BP=0.658 \left( SO \right)+186.3997$$

Enthalpy of Vaporization:

$$EV=2.5565 \left( IS \right)+20.9296$$

$$EV=15.859 \left( MIR \right)+32.2719$$

$$EV=7.9164 \left( MR \right)+33.9733$$

$$EV=2.6888 \left( MDD \right)+34.9873$$

$$EV=10.7478 \left( MHD \right)+31.6221$$

$$EV=2.1388 \left( M^{2}DR \right)+33.9811$$

$$EV=2.1827 \left( M^{2}D^{2}R \right)+23.7437$$

$$EV=1.5949 \left( M^{2}D^{2} \right)+25.0334$$

$$EV=0.7254 \left( M^{2}{SD}^{2} \right)+28.2502$$

$$EV=1.168 \left( SDD \right)+22.4092$$

$$EV=6.1456 \left( SCD \right)+23.67$$

$$EV=3.9054 (ABC)+22.66947$$

$$EV=0.5935\left( M_{1} \right)+19.4940$$

$$EV=13.1304 \left( M_{2} \right)+27.0939$$

$$EV=0.5143 \left( SZ \right)+16.4506$$

$$EV=1.1871 \left( SK \right)+19.4941$$

$$EV=1.0277 \left( {SK}_{1} \right)+17.0787$$

$$EV=2.9741 \left( GA \right)+22.2439$$

$$EV=6.2474 \left( Randic \right)+24.8865$$

$$EV=0.8098\left( HZ \right)+19.7144$$

$$EV=0.112 \left( SO \right)+17.8431$$

Flash Point:

$$FP=8.4689 \left( IS \right)+78.3803$$

$$FP=52.7018 \left( MIR \right)+114.8512$$

$$FP=26.332\left( MR \right)+120.1794$$

$$FP=8.95 \left( MDD \right)+123.3075$$

$$FP=26.3323 \left( MHD \right)+120.1794$$

$$FP=7.1255 \left( M^{2}DR \right)+119.6583$$

$$FP=7.2352 \left( M^{2}D^{2}R \right)+87.4606$$

$$FP=5.2956 \left( M^{2}D^{2} \right)+91.118$$

$$FP=2.4197 \left( M^{2}{SD}^{2} \right)+100.1323$$

$$FP=3.8727 \left( SDD \right)+82.8417$$

$$FP=20.3283 \left( SCD \right)+88.0171$$

$$FP=12.9340 (ABC)+84.3303$$

$$FP=1.9688 \left( M_{1} \right)+73.1035$$

$$FP=43.3556 \left( M_{2} \right)+99.9909$$

$$FP=1.7086 \left( SZ \right)+62.374$$

$$FP=3.9377 \left( SK \right)+73.1035$$

$$FP=3.4165 \left( {SK}_{1} \right)+64.1678$$

$$FP=9.8428 \left( GA \right)+83.0979$$

$$FP=20.6558 \left( Randic \right)+92.2075$$

$$FP=2.687 \left( HZ \right)+73.73249$$

$$FP=0.3980 \left( SO \right)+66.4949$$

Molecular Refractivity:

$$MR=2.9 \left( IS \right)+0.0112$$

$$MR=18.7248 \left( MIR \right)+11.9564$$

$$MR=12.7483+10.8183$$

$$MR=3.16 \left( MDD \right)+15.4676$$

$$MR=12.7438 \left( MHD \right)+10.8182$$

$$MR=2.4395 \left( M^{2}DR \right)+16.5815$$

$$MR=2.5390 \left( M^{2}D^{2}R \right)+1.6985$$

$$MR=1.865324 \left( M^{2}D^{2} \right)+3.1746$$

$$MR=0.8672 \left( M^{2}{SD}^{2} \right)+5.5666$$

$$MR=1.3612 \left( SDD \right)-0.1415$$

$$MR=7.2061 \left( SCD \right)+0.3023$$

$$MR=4.5464 (ABC)+0.0241$$

$$MR=0.6760 \left( M_{1} \right)-1.6899$$

$$MR=15.8226 \left( M_{2} \right)+2.06$$

$$MR=0.5764 \left( SZ \right)-5.2798$$

$$MR=1.352 \left( SK \right)-1.6899$$

$$MR=1.1457 \left( {SK}_{1} \right)-2.5516$$

$$MR=3.45 \left( GA \right)-0.4421$$

$$MR=7.3982 \left( Randic \right)+0.9284$$

$$MR=0.9236 \left( HZ \right)-1.4941$$

$$MR=0.134 \left( SO \right)-2.0407$$

Polar Surface Area :

$$PSA=4.8172 \left( IS \right)-3.1169$$

$$PSA=31.3466 \left( MIR \right)+15.002$$

$$PSA=15.5152\left( MRD \right)+19.8022$$

$$PSA=5.2589 \left( MDD \right)+22.1636$$

$$PSA=21.3704\left( MHD \right)+12.7118$$

$$PSA=3.9609 \left( M^{2}DR \right)+29.2362$$

$$PSA=4.2316 \left( M^{2}D^{2}R \right)-1.1019$$

$$PSA=3.1091 \left( M^{2}D^{2} \right)+1.3381$$

$$PSA=1.4423 \left( M^{2}{SD}^{2} \right)+5.8316$$

$$PSA=2.2672 \left( SDD \right)-4..0206$$

$$PSA=12.0431 \left( SCD \right)-4.097$$

$$PSA=7.5786(ABC)-3.9358$$

$$PSA=1.1184 \left( M_{1} \right)-4.9553$$

$$PSA=26.6984 \left( M_{2} \right)-3.4704$$

$$PSA=0.952 \left( SZ \right)-10.4498$$

$$PSA=2.2368 \left( SK \right)-4.9555$$

$$PSA=1.9278 \left( {SK}_{1} \right)-2.7093$$

$$PSA=5.7973 \left( GA \right)+1.9877$$

$$PSA=12.4 \left( Randic \right)-3.45081$$

$$PSA=1.5283\left( HZ \right)-4.67$$

$$PSA=0.2202 \left( SO \right)-3.885$$

Polarization:

$$Polariza=1.1498 \left( IS \right)-0.0036$$

$$Polariza=7.4242 \left( MIR \right)+4.7306$$

$$Polariza=3.6887 \left( MR \right)+5.6699$$

$$Polariza=1.2529 \left( MDD \right)+6.1234$$

$$Polariza=5.0529 \left( MHD \right)+4.2791$$

$$Polariza=0.9672 \left( M^{2}DR \right)+6.5623$$

$$Polariza=1.0067 \left( M^{2}D^{2}R \right)+0.6649$$

$$Polariza=0.7395 \left( M^{2}D^{2} \right)+1.2497$$

$$Polariza=0.3438 \left( M^{2}{SD}^{2} \right)+2.1971$$

$$Polariza=0.5397 \left( SDD \right)-0.0646$$

$$Polariza=2.8570 \left( SCD \right)+0.112$$

$$Polariza=1.8026 (ABC)+0.0014$$

$$Polariza=0.2680 \left( M_{1} \right)-0.67815$$

$$Polariza=6.2733 \left( M_{2} \right)+0.8088$$

$$Polariza=0.2285 \left( SZ \right)-2.1010$$

$$Polariza=0.536 \left( SK \right)-0.6782$$

$$Polariza=0.4542 \left( {SK}_{1} \right)-1.0197$$

$$Polariza=1.3906 \left( GA \right)+0.9295$$

$$Polariza=2.9332 \left( Randic \right)+0.3602$$

$$Polariza=0.3662 \left( HZ \right)-0.6$$

$$Polariza=0.0531 \left( SO \right)-0.8174$$

Molar Volume:

$$MV=7.5801\left( IS \right)+2.4761$$

$$MV=49.3926\left( MIR \right)+30.5036$$

$$MV=24.4859 \left( MRD \right)+37.5215$$

$$MV=8.3078 \left( MDD \right)+40.9086$$

$$MV=33.6529 \left( MHD \right)+27.1097$$

$$MV=6.5026\left( M^{2}DR \right)+39.1243$$

$$MV=6.6798 \left( M^{2}D^{2}R \right)+4.44571$$

$$MV=4.9107 \left( M^{2}D^{2} \right)+8.0736$$

$$MV=2.2856 \left( M^{2}{SD}^{2} \right)+13.9663$$

$$MV=3.5818 \left( SDD \right)-0.46$$

$$MV=18.9822 \left( SCD \right)+0.2898$$

$$MV=11.9619 (ABC)+0.0111$$

$$MV=1.7708 \left( M_{1} \right)-2.7954$$

$$MV=42.9079 \left( M_{2} \right)+2.8513$$

$$MV=1.5 \left( SZ \right)-9.8438$$

$$MV=3.5415 \left( SK \right)-2.7954$$

$$MV=2.9858 \left( {SK}_{1} \right)-3.0667$$

$$MV=9.0693 \left( GA \right)-0.8786$$

$$MV=19.5266\left( Randic \right)+1.1891$$

$$MV=2.42 \left( HZ \right)-2.3637$$

$$MV=0.3495 \left( SO \right)-1.954$$

**Predicted and Actual Values of Properties-Linear Regression**

| **Property** | **MW** | | **MASS** | | **TPSA** | |
| --- | --- | --- | --- | --- | --- | --- |
| **Chemical** | **Actual** | ***Predicted*** | **Actual** | ***Predicted*** | **Actual** | ***Predicted*** |
| Ceftriaxone | 554.6 | 524.683 | 554.04 | 524.215 | 288 | 207.479 |
| Aminogliycoside66_40C | 857 | 837.000 | 856.454 | 836.291 | 348 | 325.359 |
| CephalosporinC | 415.4 | 403.760 | 415.1 | 403.386 | 202 | 162.997 |
| Ertapenem | 475.5 | 464.412 | 475.14 | 463.990 | 182 | 185.238 |
| Tigecycline | 585.6 | 587.029 | 585.28 | 586.513 | 206 | 228.609 |
| Linezolid | 337.35 | 395.927 | 337.143 | 395.558 | 71.1 | 149.652 |
| Gentamicin | 477.6 | 475.499 | 477.31 | 475.069 | 200 | 190.798 |
| Daptomycin | 1620.7 | 1623.545 | 1619.71 | 1622.229 | 702 | 678.996 |
| Chloramphenicol | 323.13 | 289.538 | 322.01 | 289.252 | 115 | 119.997 |
| Tedizolid | 370.3 | 401.251 | 370.12 | 400.878 | 106 | 154.100 |
| Doxycycline | 444.4 | 488.269 | 444.15 | 487.829 | 182 | 198.583 |
| Cefotaxime | 455.5 | 437.436 | 455.05 | 437.036 | 227 | 174.859 |
| Impinenem | 299.35 | 293.268 | 299.09 | 292.979 | 142 | 118.514 |
| Meropenem | 383.5 | 373.945 | 383.15 | 373.594 | 136 | 145.945 |
| Vancomycin | 1449.2 | 1450.821 | 1447.43 | 1449.638 | 531 | 574.091 |
| Piperacillin | 517.6 | 520.346 | 517.16 | 519.881 | 182 | 204.885 |

| **Property** | **COM** | | **MR** | | **PSA** | |
| --- | --- | --- | --- | --- | --- | --- |
| **Chemical** | **Actual** | **Predicted** | **Actual** | **Predicted** | **Actual** | **Predicted** |
| Ceftriaxone | 1110 | 853.8244 | 130 | 127.558 | 288 | 190.799 |
| Aminogliycoside66_40C | 1500 | 1598.3421 | 196.3 | 203.750 | 348 | 326.146 |
| CephalosporinC | 737 | 736.2691 | 96 | 98.058 | 202 | 169.429 |
| Ertapenem | 893 | 736.2691 | 118.3 | 112.854 | 182 | 169.429 |
| Tigecycline | 1240 | 1363.231 | 151.3 | 142.768 | 206 | 283.405 |
| Linezolid | 472 | 422.7882 | 83 | 96.147 | 71 | 112.441 |
| Gentamicin | 636 | 932.1947 | 122.6 | 115.559 | 200 | 205.046 |
| Daptomycin | 3480 | 3322.487 | 399.3 | 395.635 | 702 | 639.579 |
| Chloramphenicol | 342 | 540.3436 | 72.6 | 70.192 | 115 | 133.811 |
| Tedizolid | 543 | 501.1584 | 93.6 | 97.446 | 106 | 126.688 |
| Doxycycline | 956 | 1167.305 | 109 | 118.674 | 182 | 247.787 |
| Cefotaxime | 833 | 697.0840 | 106 | 106.273 | 227 | 162.305 |
| Impinenem | 491 | 383.6030 | 72.7 | 71.102 | 139 | 105.317 |
| Meropenem | 679 | 697.0840 | 96.8 | 90.784 | 135 | 162.305 |
| Vancomycin | 2960 | 3009.006 | 350.8 | 353.498 | 530 | 582.591 |
| Piperacillin | 982 | 893.0096 | 128.5 | 126.500 | 182 | 197.923 |

| **Property** | **POLAR** | | **MV** | | **BP** | |
| --- | --- | --- | --- | --- | --- | --- |
| **Chemical** | **Actual** | **Predicted** | **Actual** | **Predicted** | **Actual** | **Predicted** |
| Ceftriaxone | 51.5 | 50.566 | 281.7 | 333.458 | ------ | ------ |
| Aminogliycoside66_40C | 77.8 | 80.775 | 491.1 | 518.551 | 1109.1 | 1123.875 |
| CephalosporinC | 38.1 | 38.870 | 266.6 | 263.611 | 814.7 | 687.804 |
| Ertapenem | 46.9 | 44.736 | 306.2 | 298.535 | 813.9 | 747.540 |
| Tigecycline | 60 | 56.597 | 402.5 | 366.635 | 890.9 | 864.025 |
| Linezolid | 32.9 | 38.112 | 259 | 242.658 | 585.5 | 651.963 |
| Gentamicin | 48.6 | 45.809 | 366.9 | 307.266 | 669.4 | 762.474 |
| Daptomycin | 158.3 | 156.853 | 1110.8 | 1073.831 | 2078.2 | 2073.673 |
| Chloramphenicol | 28.8 | 27.822 | 208.8 | 196.093 | 644.9 | 572.315 |
| Tedizolid | 37.1 | 38.627 | 235.6 | 249.642 | 614.5 | 663.910 |
| Doxycycline | 43.2 | 47.044 | 271.1 | 319.489 | 762.6 | 783.381 |
| Cefotaxime | 42 | 42.127 | 252.8 | 282.237 | ------ | ------ |
| Impinenem | 28.8 | 28.183 | 183.9 | 193.765 | 530.2 | 568.333 |
| Meropenem | 38.4 | 35.986 | 268.9 | 236.837 | 627.4 | 642.007 |
| Vancomycin | 139.1 | 140.146 | 874.7 | 909.109 | ------ | ------ |
| Piperacillin | 50.9 | 50.147 | 340.5 | 329.384 | ------ | ------ |

| **Property** | **EV** | | **FP** | |
| --- | --- | --- | --- | --- |
| **Chemical** | **Actual** | **Predicted** | **Actual** | **Predicted** |
| Ceftriaxone | ------ | ------ | ------ | ------ |
| Aminogliycoside66_40C | 184.4 | 188.6711 | 624.6 | 633.505 |
| CephalosporinC | 128.7 | 108.7943 | 446.5 | 369.759 |
| Ertapenem | 124 | 119.7364 | 446 | 405.888 |
| Tigecycline | 135.7 | 141.0733 | 492.6 | 476.341 |
| Linezolid | 87.5 | 102.2291 | 307.9 | 348.081 |
| Gentamicin | 112.6 | 122.4719 | 358.6 | 414.921 |
| Daptomycin | 366.9 | 362.6493 | 1210.7 | 1207.966 |
| Chloramphenicol | 100 | 87.63976 | 343.8 | 299.908 |
| Tedizolid | 95.9 | 104.4175 | 325.4 | 355.307 |
| Doxycycline | 116.5 | 126.3016 | 415 | 427.566 |
| Cefotaxime | ------ | ------ | ------ | ------ |
| Impinenem | 92.7 | 86.9103 | 274.5 | 297.499 |
| Meropenem | 106.4 | 100.4054 | 333.2 | 342.059 |
| Vancomycin | ------ | ------ | ------ | ------ |
| Piperacillin | ------ | ------ | ------ | ------ |

**Predicted and Actual Values of Properties-Quadratic Regression:**

| **Properties** | **MW** | | **Mass** | | **MR** | |
| --- | --- | --- | --- | --- | --- | --- |
| **Phytochemical** | Actual | *Predicted* | **Actual** | *Predicted* | Actual | *Predicted* |
| Ceftriaxone | 554.6 | 501.242 | 554.04 | 500.8632 | 130 | 121.472 |
| Aminoglycoside 66-40C | 857 | 826.677 | 856.454 | 826.2 | 196.3 | 200.099 |
| cephalosporin C | 415.4 | 403.713 | 415.1 | 403.312 | 96 | 98.171 |
| Ertapenem | 475.5 | 451.202 | 475.14 | 450.816 | 118.3 | 109.5 |
| Tigecycline, (4R)- | 585.6 | 627.652 | 585.28 | 627.264 | 151.3 | 151.865 |
| Linezolid | 337.35 | 384.975 | 337.143 | 384.566 | 83 | 93.711 |
| Gentamicin | 477.6 | 474.889 | 477.31 | 474.507 | 122.6 | 115.162 |
| Daptomycin | 1620.7 | 1589.898 | 1619.71 | 1588.461 | 399.3 | 388.212 |
| Chloramphenicol | 323.13 | 303.977 | 322.01 | 303.523 | 72.6 | 74.497 |
| Tedizolid | 370.3 | 384.975 | 370.12 | 384.566 | 93.6 | 93.711 |
| Doxycycline | 444.4 | 492.292 | 444.15 | 491.913 | 109 | 119.328 |
| Cefotaxime | 455.5 | 424.178 | 455.05 | 423.785 | 106 | 103.049 |
| Imipenem | 299.35 | 303.977 | 299.09 | 303.523 | 72.7 | 74.497 |
| Meropenem | 383.5 | 392.764 | 383.15 | 392.359 | 96.8 | 95.565 |
| Vancomycin | 1449.2 | 1488.778 | 1447.43 | 1487.513 | 350.8 | 363.061 |
| Piperacillin | 517.6 | 515.544 | 517.16 | 515.166 | 128.5 | 124.9 |

| **Properties** | **POLAR** |  | **MV** |  | **EV** |  |
| --- | --- | --- | --- | --- | --- | --- |
| **Phytochemical** | **Actual** | **Predicted** | **Actual** | **Predicted** | **Actual** | **Predicted** |
| Ceftriaxone | 51.5 | 48.148 | 281.7 | 293.600 | -------- | ---------- |
| Aminoglycoside 66-40C | 77.8 | 79.313 | 491.1 | 468.127 | 184.4 | 187.178 |
| cephalosporin C | 38.1 | 38.917 | 266.6 | 270.514 | 128.7 | 104.740 |
| Ertapenem | 46.9 | 43.405 | 306.2 | 270.514 | 124 | 110.443 |
| Tigecycline, (4R)- | 60 | 60.193 | 402.5 | 392.975 | 135.7 | 138.917 |
| Linezolid | 32.9 | 37.15 | 259 | 229.429 | 87.5 | 107.208 |
| Gentamicin | 48.6 | 45.648 | 366.9 | 308.58 | 112.6 | 112.518 |
| Daptomycin | 158.3 | 153.92 | 1110.8 | 1057.459 | 366.9 | 365.909 |
| Chloramphenicol | 28.8 | 29.538 | 208.8 | 241.157 | 100 | 100.642 |
| Tedizolid | 37.1 | 37.15 | 235.6 | 241.892 | 95.9 | 106.272 |
| Doxycycline | 43.2 | 47.299 | 271.1 | 355.929 | 116.5 | 109.86 |
| Cefotaxime | 42 | 40.849 | 252.8 | 265.928 | ------ | ------- |
| Imipenem | 28.8 | 29.538 | 183.9 | 219.288 | 92.7 | 101.191 |
| Meropenem | 38.4 | 37.884 | 268.9 | 263.26 | 106.4 | 106.423 |
| Vancomycin | 139.1 | 143.942 | 874.7 | 942.183 | -------- | -------- |
| Piperacillin | 50.9 | 49.507 | 340.5 | 300.263 | --------- | --------- |

**Predicted and Actual Values of Properties-Cubic Regression:**

| **Property** | **COM** | | **MR** | | **POLARIZATION** | | **MV** | |
| --- | --- | --- | --- | --- | --- | --- | --- | --- |
| **Chemical** | **Actual** | ***Predicted*** | **Actual** | ***Predicted*** | **Actual** | ***Predicted*** | **Actual** | ***Predicted*** |
| Ceftriaxone | 1110 | 950.424 | 130 | 124.214 | 51.5 | 49.233 | 281.7 | 327.161 |
| Aminogliycoside66_40C | 1500 | 1495.288 | 196.3 | 191.981 | 77.8 | 76.101 | 491.1 | 458.238 |
| CephalosporinC | 737 | 700.141 | 96 | 99.346 | 38.1 | 39.381 | 266.6 | 267.523 |
| Ertapenem | 893 | 830.605 | 118.3 | 111.918 | 46.9 | 44.362 | 306.2 | 298.796 |
| Tigecycline | 1240 | 1194.554 | 151.3 | 152.167 | 60 | 60.313 | 402.5 | 384.402 |
| Linezolid | 472 | 643.444 | 83 | 94.103 | 32.9 | 37.304 | 259 | 253.799 |
| Gentamicin | 636 | 889.366 | 122.6 | 117.848 | 48.6 | 46.711 | 366.9 | 312.747 |
| Daptomycin | 3480 | 3431.115 | 399.3 | 393.786 | 158.3 | 156.125 | 1110.8 | 1066.798 |
| Chloramphenicol | 342 | 357.626 | 72.6 | 69.218 | 28.8 | 27.450 | 208.8 | 183.464 |
| Tedizolid | 543 | 643.444 | 93.6 | 94.103 | 37.1 | 37.304 | 235.6 | 253.799 |
| Doxycycline | 956 | 930.169 | 109 | 122.078 | 43.2 | 48.387 | 271.1 | 322.388 |
| Cefotaxime | 833 | 758.576 | 106 | 104.884 | 42 | 41.575 | 252.8 | 281.583 |
| Impinenem | 491 | 357.626 | 72.7 | 69.218 | 28.8 | 27.450 | 183.9 | 183.464 |
| Meropenem | 679 | 667.399 | 96.8 | 96.303 | 38.4 | 38.176 | 268.9 | 259.607 |
| Vancomycin | 2960 | 3022.396 | 350.8 | 358.057 | 139.1 | 141.962 | 874.7 | 932.783 |
| Piperacillin | 982 | 981.825 | 128.5 | 127.576 | 50.9 | 50.566 | 340.5 | 334.547 |
